# Radial and tangential migration of telencephalic somatostatin neurons originated from the mouse diagonal area

**DOI:** 10.1007/s00429-015-1086-8

**Published:** 2015-07-19

**Authors:** Luis Puelles, N. Morales-Delgado, P. Merchán, B. Castro-Robles, M. Martínez-de-la-Torre, C. Díaz, J. L. Ferran

**Affiliations:** Department of Human Anatomy and Psychobiology, School of Medicine, University of Murcia, Murcia, E30071 Spain; Department of Medical Sciences, School of Medicine, University of Castilla-La Mancha, Albacete, E02006 Spain; Division of Developmental Biology, Perinatal Institute, Cincinnati Children’s Hospital Medical Center, University of Cincinnati College of Medicine, Cincinnati, OH 45229 USA; Department of Experimental Medicine, Laboratory of Brain Development and Evolution, Institute of Biomedical Research of Lleida, University of Lleida, Lleida, Spain; Department of Human Anatomy and Psychobiology, Faculty of Medicine, University of Murcia, 30100 Murcia, Spain

**Keywords:** Forebrain interneurons, Secondary prosencephalon, Subpallium, Pallidum, Medial ganglionic eminence, Preoptic area, Entopeduncular area, Striatum, Cortex

## Abstract

The telencephalic subpallium is the source of various GABAergic interneuron cohorts that invade the pallium via tangential migration. Based on genoarchitectonic studies, the subpallium has been subdivided into four major domains: striatum, pallidum, diagonal area and preoptic area (Puelles et al. 2013; Allen Developing Mouse Brain Atlas), and a larger set of molecularly distinct progenitor areas (Flames et al. 2007). Fate mapping, genetic lineage-tracing studies, and other approaches have suggested that each subpallial subdivision produces specific sorts of inhibitory interneurons, distinguished by differential peptidic content, which are distributed tangentially to pallial and subpallial target territories (e.g., olfactory bulb, isocortex, hippocampus, pallial and subpallial amygdala, striatum, pallidum, septum). In this report, we map descriptively the early differentiation and apparent migratory dispersion of mouse subpallial somatostatin-expressing (*Sst*) cells from E10.5 onward, comparing their topography with the expression patterns of the genes *Dlx5*, *Gbx2*, *Lhx7*-*8*, *Nkx2.1*, *Nkx5.1* (*Hmx3*), and *Shh*, which variously label parts of the subpallium. Whereas some experimental results suggest that *Sst* cells are pallidal, our data reveal that many, if not most, telencephalic *Sst* cells derive from de diagonal area (Dg). *Sst*-positive cells initially only present at the embryonic Dg selectively populate radially the medial part of the bed nucleus striae terminalis (from paraseptal to amygdaloid regions) and part of the central amygdala; they also invade tangentially the striatum, while eschewing the globus pallidum and the preoptic area, and integrate within most cortical and nuclear pallial areas between E10.5 and E16.5.

**Electronic supplementary material:**

The online version of this article (doi:10.1007/s00429-015-1086-8) contains supplementary material, which is available to authorized users.

## Introduction

The identity of neuron types produced within a specific brain region is the result of progressive patterning and consequent fate specification of progenitors during early ontogeny. Establishment of a unique molecular profile at a given progenitor domain allows it to generate a particular neuronal cell type (or several of them, either sequentially, or in a salt-and-pepper pattern). Such neurons are presumed to be different at least in some subtle aspects from those produced in adjoining areas, irrespective that some part of the respective molecular profiles may be shared. Some neuronal derivatives aggregate radially within the local mantle zone, whereas others may migrate tangentially into neighbouring or distant brain areas. The latter is a well-known phenomenon in the telencephalon, where various subpallial cell populations migrate into other parts of the subpallium or into the pallium, contributing diverse contingents of inhibitory interneurons to local circuitry (reviewed in Marín and Rubenstein 2003; Gelman et al. 2009, 2012; Marin 2013). In this report, we examine areally restricted subpallial production, and subsequent migratory dispersion, of telencephalic somatostatin (Sst) neurons into various subpallial and pallial target domains, highlighting their participation in the radial development of the medial bed nucleus striae terminalis, and the lateral part of the central amygdalar nucleus (areas whose development was hitherto largely obscure).

**Fig. 1:**
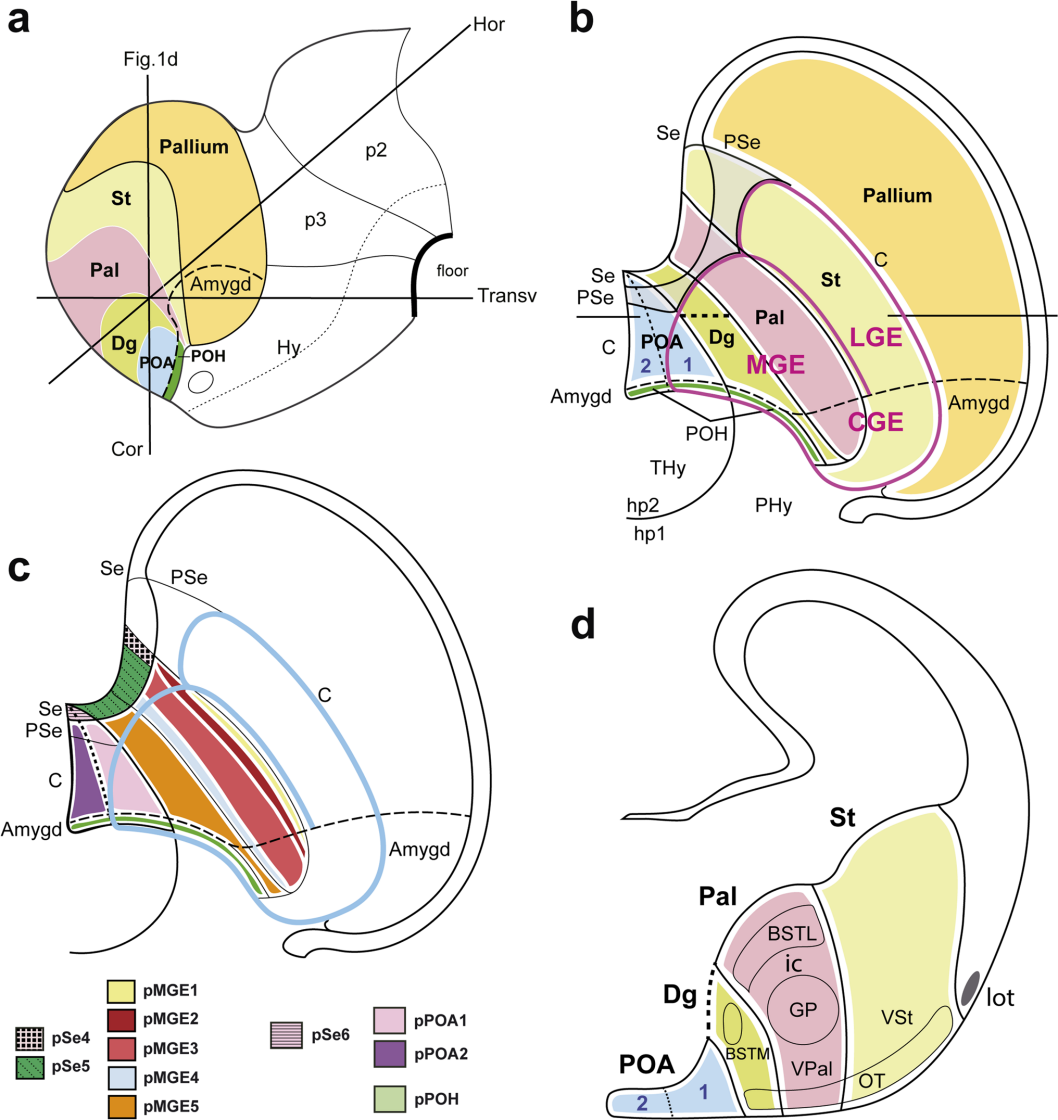
- Schemas illustrating the relative topography of subpallial subdivisions. **a** Schema of a *left-side* view of the embryonic brain, indicating in *colors* the telencephalic region. The pallium (*orange*) is separated from the subpallium by a *black line*. The subpallium appears divided into four domains: striatum (*yellow* St), pallidum (*pink* Pal), diagonal area (*green* Dg), and preoptic area (*blue* POA). The preopto-hypothalamic area (*dark green* POH), a part of POA, abbuts the telencephalic/preoptic border with respect to the terminal and peduncular parts of the hypothalamus (THy, PHy); The POH is continuous laterally across the hemispheric stalk with the subpallial and pallial amygdala (all three enclosed by the *dash line*). The longitudinal alar/basal boundary of the forebrain is depicted as a *dotted line*; the forebrain floor plate is marked by a *thick black line*; p2–p3 refer to diencephalic prosomeres. The coronal (Cor), horizontal (Hor), and transverse (Transv) planes of section used in this study are indicated; note the Hor and Transv planes are oriented relative to the prosomeric length axis and floor plate, while the Cor plane is arbitrary, and corresponds to the coronal section schema in (**d**). **b** Two-dimensional schema looking down on a flattened view of the right hemisphere, in which the four subpallial domains are mapped relative to the septal region, the hypothalamus, the pallium, and the medial, lateral and caudal ganglionic eminences (*red contour*; MGE, LGE, CGE; note the mixed composition of the MGE). The color code, areal names, and dash line used in (**a**) apply here to the ‘central’ or principal region of the subpallium in (**b**). The septo-amygdaloid axis can be imagined, with an obliquity that characterizes particularly the evaginated subpallial domains (St, Pal; less so Dg, or POA). The septal end of the subpallium (Se) is strictly *septal*, and contains in principle the topological *dorsal* end of all four subpallial domains (note the commissural septal midline lies at the telencephalic roof plate). Intercalated between the *septal* (Se) and the *central* subpallial sectors (C) there appears the *paraseptal* subpallial sector (PSe), which connects them (passing under the interventricular foramen), whereas the subpallial amygdala found within the CGE represents the *amygdaloid* subpallial sector (Amygd). The four main subpallial domains thus stretch from the septum into the amygdala. The Dg domain, of particular interest in the present study, lies precisely at the hemispheric stalk. The level of the schematic coronal section shown in (**d**) is indicated. **c** This schema is basically a reproduction of (**b**), used for tentative mapping of the Se, MGE, and POA progenitor domains distinguished by Flames et al. (2007) relative to the ganglionic eminences (*blue contour line*); the color code indicated for these areas is slightly modified (for visibility) from that used by these authors; note the LGE progenitor areas are not represented (not needed in the present context). **d** Conventional schema of a coronal cross section through the telencephalon, in which the central subpallial domains are intersected side by side—see section plane in (**a**) and (**b**). Comparison of the dashed ventricular contour of the sectioned Dg (pMGE5) area with the *dash line* in (**b**) illustrates our idea that, in three dimensions, this domain is not a localized neuroepithelial patch, but an obliquely elongated band. POA and Dg converge rostrodorsally at the crossing of the anterior commissure (septocommissural preoptic area)

We presently understand the subpallium as consisting of four main partitions stretched along the septoamygdaloid axis; these are, from medial to lateral: *preoptic area* (POA), *diagonal area* (Dg), *pallidum* (Pal), and *striatum* (St) (Fig. 1a, b; Allen Developing Mouse Brain Atlas; Medina and Abellan 2012; Puelles et al. 2013). Historically, the Dg was first identified as anterior entopeduncular area (AEP; Bulfone et al, 1993; Puelles and Rubenstein, 1993; Rubenstein et al. 1994). This is a somewhat misleading and unsatisfactory term, since it refers exclusively to an intrapeduncular locus and not to a full histogenetic domain. Therefore, it was later substituted by some authors by rough topographic reference to the part of the MGE (caudoventral, caudomedial or ventral) occupied by this domain—cvMGE/cmMGE/vMGE; separately, Flames et al. (2007) identified the corresponding progenitor domain as pMGE5 (see Fig. 1c). Finding all these names anatomically imprecise and not distinctive enough, Puelles proposed the diagonal area (Dg) name while working on the terminology used in the Allen Developing Mouse Brain Atlas (developingmouse.brain-map.org; online since 2009; Puelles et al, 2013). This name refers to the inclusion within the referred histogenetic domain of the classical diagonal band nuclei and the related substantia innominata. These landmarks allow easy anatomic identification of the Dg with regard to Pal and POA. A comparable set of four areal subdivisions (*septal, paraseptal, central, and amygdaloid*) can be distinguished generically across each of these main domains, forming parallel series along the septoamygdaloid axis (Fig. 1b). These subareas were systematized in the Allen Developing Mouse Brain Atlas (http://www.developingmouse.brain-map.org), as well as by Puelles et al. (2013). The central parts of POA, Dg, and Pal participate in the MGE, whereas the central St occupies most of the LGE (Fig. 1b; Flames et al. 2007). The corresponding paraseptal parts (e.g., nucleus accumbens) are found rostromedially, at the locus where the POA, Dg, Pal, and St areas extend under the lateral ventricle and the interventricular foramen into the medial septal wall; septal subdivisions corresponding to the POA, Dg, Pal, and St domains can be identified as well (Fig. 1b; Puelles et al. 2000, 2004; Flames et al. 2007). At the opposite end of the septoamygdaloid axis, amygdaloid parts of St, Pall, and Dg conform the CGE, which is also medially continuous with the preopto-hypothalamic transition area; the latter may be added to the extended amygdala concept (POH; Fig. 1b).

The molecular phenotype of the subpallial ventricular and subventricular zone was used by Flames et al. (2007) to define 18 molecularly distinct progenitor areas, mapped to the septum, ganglionic eminences, and preoptic area; part of these areas are represented in our Fig. 1c (pLGE1-4, not shown; pMGE1-5; pPOA1,2, pPOH, pSe1-6).

Most neurons generated from these different subpallial progenitor areas are GABAergic. Some of them settle radially into the local mantle and differentiate as projection neurons or interneurons. In addition, groups of GABAergic and cholinergic neurons migrate tangentially from some subpallial areas into other subpallial areas (Marín and Rubenstein 2003; Gelman et al. 2009, 2012; Marin 2013). Other GABAergic interneurons of various subpallial origins migrate into the pallium (both cortex and nuclei), constituting in the adult roughly 20 % of the total cortical population. The elements that migrate into the cortex have been classified into three or four non-overlapping populations expressing either parvalbumin (PV), somatostatin (SST), or calretinin/vasointestinal peptide (CR/VIP), with the possible addition of neuropeptide Y (NPY/reelin) cells (Marin and Rubenstein 2001, 2003; Wonders and Anderson 2006; Gelman and Marin 2010; Miyoshi et al. 2010; Xu et al. 2010; Lee et al. 2010).

### Somatostatin neurons in the telencephalon

The hormone/neuropeptide somatostatin (SST; also known as somatotropin-release inhibiting factor) was first isolated from hypothalamic extracts on the basis of its ability to inhibit growth hormone secretion from the anterior pituitary (Brazeau et al. 1973). The somatostatin mRNA precursor is translated to produce a large inactive pre-pro-somatostatin peptide (116 amino acids; PPSST); its post-translational enzymatic cleavage yields two biologically active products, somatostin 14 (14 amino acids; SST-14) and somatostin 28 (28 amino acids; SST-28), which have neurotransmitter and neuromodulator roles (Kumar and Grant 2010). SST is known to be involved in granule cell migration during cerebellar development (Epelbaum et al. 1994; Yacubova and Komuro 2002; Le Verche et al. 2009). Several mapping studies performed in embryonic, postnatal, and adult mice showed that SST has a wide central nervous system distribution that includes cerebral cortex, hippocampus, striatum, amygdala, olfactory system, hypothalamus, diencephalon, midbrain, and brainstem (Roberts et al. 1982, Moga and Gray 1985, Gray and Magnuson 1992; Garcia-Lopez et al. 2008; Viollet et al. 2008; Real et al. 2009; Bupesh et al. 2011a, b; Morales-Delgado et al. 2011).

According to in vitro and in vivo fate-mapping studies, the medial ganglionic eminence (MGE), which is the sum of the central parts of Pal, Dg and POA (Fig. 1b), is currently conceived as the main source of PV+ and SST+ cortical interneurons (Xu et al. 2004; Butt et al. 2005; Fogarty et al. 2007; Ghanem et al. 2007). However, there is so far no consensus on the specific origin of SST neurons within the MGE. The ‘dorsal’ MGE area (which includes mainly the pMGE1 subdomain of Flames et al. 2007, which differentially expresses *Nkx6.2*) was specifically proposed as the main source of *Sst*-expressing cortical interneurons by Wonders et al. (2008), a conclusion that was supported by other genetic lineage tracings done in mice (e.g., Sousa et al. 2009). However, lineage studies using *Nkx2.1Cre* labeling suggested that most SST+ cells derive from the ‘central and ventral MGE’ subregion, which corresponds roughly to the pMGE5 area of Flames et al. 2007 (Fogarty et al. 2007; Xu et al. 2008). An area that seems likewise to correspond to pMGE5, but that was referred to as ‘caudal and medial MGE’, was reported to be a source of calbindin-containing neurons that enter the pallial amygdala (Neri et al. 2002; Legaz et al. 2005). Several reports of Medina and collaborators concluded that the ‘caudoventral MGE’ (presumably still the same area), contributes SST+ interneurons to the amygdala (García-López et al. 2008; Bupesh et al. 2011a, b; Medina and Abellan 2012; see also Real et al. 2009). We hold that these positional terms (caudoventral/caudomedial MGE) all essentially refer to the Dg domain of our terminology, which we conceive as elongated along the septoamygdaloid axis (Fig. 1b), whereas the cited authors seem to think of a more circumscribed neuroepithelial patch. Most of the cited studies using transgenic mice analyzed their data from E12.5 or E13.5 onward, whereas the earliest subpallial *Sst* cells appear at E10.5 (present results). The present full descriptive data accordingly provide information about the appropriateness of E12.5/E13.5 material for the deductions obtained from those transgenic experiments.

In the present work, we used the updated model of subpallial areal subdivisions (Fig. 1) to analyze in detail the spatiotemporal distribution of *Sst* mRNA expression during early development in the mouse telencephalon, aiming to trace overall developmental distribution of this cell type in the telencephalon, starting at initial stages. In order to illuminate the issue of a potential localized source within a subdomain of the MGE (the dorsal Pal, or pMGE1, and the Dg, or pMGE5, as suggested alternative candidates), *Sst* mRNA expression was compared in adjacent sections with *Shh* signal and several differentially expressed transcription factors (*Dlx5*, *Gbx2*, *Lhx7*-*8*, *Nkx2.1*, *Nkx5.1*) over the embryonic period E9.5 to E16.5. Our study led us to pinpoint the Dg domain as the first subpallial domain that contains cells expressing *Sst* in its mantle (from E10.5 onwards). At this time point, these diagonal *Sst* cells clearly are topographically distinct from pallidal *Gbx2*-positive cells, as well as from preoptic *Shh*/*Nkx5.1*-positive derivatives. At E12.5, many *Sst* cells apparently derived from the Dg domain have already invaded tangentially the striatal mantle (traversing subpially the pallidal domain, but clearly eschewing its central mantle) and start to migrate subpially past the LGE into the pallium. The tangentially migrated *Sst* population increases markedly subsequently, but no other locus (in dorsal Pal or elsewhere) was found outside the Dg where *Sst* cells clearly seem to arise from the ventricular or subventricular zone. However, our material may not be sufficient to negate altogether that possibility, since the expression of *Sst* may start after some delay. Our analysis accordingly suggests that numerous *Sst*-expressing neurons that colonize cortical and subcortical structures seem to derive *tangentially* from the Dg domain. Our data indicate that the *Sst*-positive components that eventually populate the medial bed nucleus striae terminalis complex and a part of the central amygdala represent *radial* derivatives of the Dg, produced along the length of its septoamygdaloid dimension.

## Materials and methods

All experimental procedures involving use and care of laboratory animals were conducted in compliance with the current normative standards of the European Community (86/609/EEC) and the Spanish Government (Royal Decree, 1201/2005; Law 32/2007).

### Animals and tissue preparation

For the present research, *Swiss albino* mouse embryos were collected from embryonic day (E) 9.5–16.5 after fertilization (adult specimens were collected as well). Noon on the day of the appearance of the vaginal plug was considered day 0.5 of gestation (E0.5). Mouse embryos were separately staged according to the Theiler stages (TS; Theiler 1989). For every embryonic age, we examined three to five mouse embryos. Timed-pregnant dams were sacrificed by cervical dislocation and embryos were immediately removed by cesarean section, anesthetized by cold, and decapitated. The heads were immersion-fixed in freshly made 4 % paraformaldehyde in 0.1 M phosphate-buffered saline (PBS, pH 7.4). The adult specimens were perfused under anesthesia with the same solution. The brains were then dissected out and post-fixed overnight at 4 °C in the same fixative. For cryostat sections, embryonic brains were first transferred to 30 % sucrose in 0.1 M PBS for 24 h at 4 °C, and then placed in 15 % gelatin/20 % sucrose solution at 37 °C until they sank. They were next embedded in the same solution, hardening the blocks at 4 °C. These primary blocks were subsequently trimmed in order to establish the desired sectioning plane, and were then frozen for 2 min in isopentane cooled to −55 °C in dry ice, and either kept frozen for future use, or placed in proper orientation upon the cryostat chuck. Sections were obtained serially 16–20 μm-thick in either the sagittal or transverse planes through the secondary prosencephalon on a Leica CM3500 S cryostat, and mounted as 3–4 parallel series onto Superfrost-plus slides (Menzel-Gläser, Braunschweig, Germany). These were stored at −20 °C until they were processed for in situ hybridization or immunohistochemistry. Some brains, including the adult ones, were embedded in 4 % low-melting point agarose (Pronadisa, Torrejón de Ardoz, Madrid, Spain, Cat. 8008), cut on a Leica VT1000 S vibratome 90 μm-thick in the sagittal or coronal planes, and processed as free-floating sections (Ferran et al. 2015a, b).

### RT-PCR

*Lhx8* and *Nkx5.1 (Hmx3)* cDNA fragments were obtained by reverse transcription (RT). RNA was individually extracted with Trizol reagent (Invitrogen, Carlsbad, CA, Cat. 10296-028) from freshly dissected brains of *Mus musculus* embryos at E10.5, E12.5, and E14.5. The RNA was treated with DNase I (Invitrogen, Cat. 18068-015) for 15 min at room temperature (RT), and the enzyme was then inactivated at 65 °C. Afterward, RNA samples were converted to single-stranded cDNA with Superscript III reverse transcriptase (Invitrogen, Cat. 18080-044) and oligo-dT-anchored primers. The resulting first-strand cDNA (0.5 μl of the reverse transcription reaction) was used as a template for the PCR reaction, which was performed in presence of *Taq* polymerase (Promega, Cat. M8305) and the following gene-specific primers for *Lhx8* and *Nkx5.1 (Hmx3)* mRNA.

- mLhx8F: 5′-AGCTGGTATGTGACGAGCA-3′
- mLhx8R: 5′-AGAATGGTTGGGACTGACG-3′
- mNkx5.1F: 5′-GACCACAAGGAGCTGGACTC-3′
- mNkx5.1R: 5′-TAAGAGGAGAAGCGCCTCAA-3′

The PCR conditions used were an initial denaturation step at 94 °C for 5 min, then 35 cycles [30 s at 94 °C, plus 1 min at Tm temperature (58 °C), and 1 min at 72 °C], followed by 20 min at 72 °C. The PCR products were cloned into the pGEM-T Easy Vector (Promega, Cat. A1360), and sequenced (SAI, University of Murcia).

### In situ hybridization

The embryos were processed for in situ hybridization with digoxigenin-UTP-labeled antisense riboprobes. Sense and antisense digoxigenin-labeled riboprobes for mouse *Dlx5*, *Gbx2*, *Lhx8*, *Nkx2.1, Nkx5.1,Shh*, and *Sst* were synthetized with a kit, following the manufacter´s recommendations (Roche Diagnostics S.L. Applied Science, Barcelona, Spain), and applying specific polymerases (Fermentas, Madrid, Spain). Plasmid information is provided in Table 1. In situ hybridization on cryosections was performed basically as described by Ferran et al. (2015a, b). Sections were not treated with proteinase K before prehybridization. Hybridizations were carried out overnight at 72 °C. Hybridization experiments on floating sections were performed following the protocol described by Ferran et al. (2015b). After hybridization, all sections were washed and incubated in a solution containing alkaline phosphatase-coupled anti-digoxigenin antibody (diluted 1:3.500; Roche Diagnostics). Nitroblue tetrazolium/5-bromo-4-chloro-3-indolyl phosphate (NBT/BCIP; Roche) solution was then used as chromogenic substrate for the final alkaline phosphatase reaction (Boehringer, Mannheim, Germany). No specific signal was obtained with sense probes (data not shown). To identify the diverse telencephalic cell masses during forebrain development, we consulted atlases of the developing mouse brain (e.g., Allen Developing Mouse Brain Atlas, http://www.developingmouse.brain-map.org), as well as our own previously published studies on the subject.

**Table 1:** List of the gene probes used for ISH and their principal characteristics

Gene symbol	NCBI accession no.	Size (bp)	Position	Linearization enzyme/polymerase	Publication/Laboratory
*Dlx5*	NM_010056.2	1180	106–1285	NcoI/Sp6	Morales-Delgado et al. 2011
*Gbx2*	NM_010262.3	1040	422–1461	HindIII/T7	Martínez S. lab
*Lhx7*-*8*	NM_010713.2	963	176–1138	SacII/Sp6	Present results
*Nkx2.1*	NM_009385.2	2216	597–2813	SalI/T3	Rubenstein J.L.R. lab
*Nkx5.1*	NM_008257.2	785	487–1271	SphI/Sp6	Present results
*Otp*	NM_011021.2	412	179–592	EcoR1/Sp6	Morales-Delgado et al. 2011
*Shh*	NM_009170.2	643	442–1084	HindIII/T3	McMahon A. lab
*Sst*	NM_009215	556	6–561	NdeI/T7	Morales-Delgado et al. 2011

### Immunohistochemistry

Our immunohistochemical reaction protocol has been described in detail elsewhere (Bardet et al. 2006; Ferran et al. 2015b). Rabbit polyclonal antiserum against rat NKX2.1 and monoclonal antiserum against rat tyrosine hydroxilase were diluted 1:1000 for use (anti-thyroid transcription factor 1 or TTF-1; Biopat Immunotechnologies, Caserta, Italy; no. PA 0100; anti-TH, Diaserin, Stillwater, MN, USA). After washes, the sections were incubated with biotinylated goat anti-rabbit or goat anti-mouse (Vector Laboratories, CA, USA; used at 1:200 dilution) followed by a streptavidin–peroxidase complex (Vectastain-ABC kit; Vector Laboratories; 0.001 % dilution), applied for 1 h at room temperature. Peroxidase activity was developed with 0.03 % 3,3′-diaminobenzidine (Sigma; St Louis; MO, USA), plus 0.003 % hydrogen peroxidase. After immunohistochemical and hybridization labeling, the slides were washed several times in PBS, air dried and coverslipped with Cytoseal 60 (Thermo Scientific, Ref. 8310-16) or Mowiol (Calbiochem, Bad Soden, Germany, Ref. 475904). We verified the specificity of the antibodies by performing parallel control experiments that omitted the primary antibody, checking that no residual immunostaining was detected (data not shown).

### Imaging

Whole-slide digital images were acquired with a ScanScope CS digital slide scanner at high resolution (Aperio Technologies, Inc.; Vista, CA, USA). After scanning, the visualization and capture of images of adjacent labeled sections were carried out by using the Aperio software ImageScope. The images were corrected for contrast, focus, and brightness. In order to compare different gene expression patterns, the images of adjacent sections reacted with different markers were superposed and artificially pseudocolored (from blue to red or green) with Photoshop CS3. The plates were labeled using Adobe Photoshop Illustrator CS2 (Adobe Systems Inc., San José, CA, USA).

## Results

### Telencephalic *Sst* mRNA expression in the subpallium starts at E10.5

**Fig. 2:**
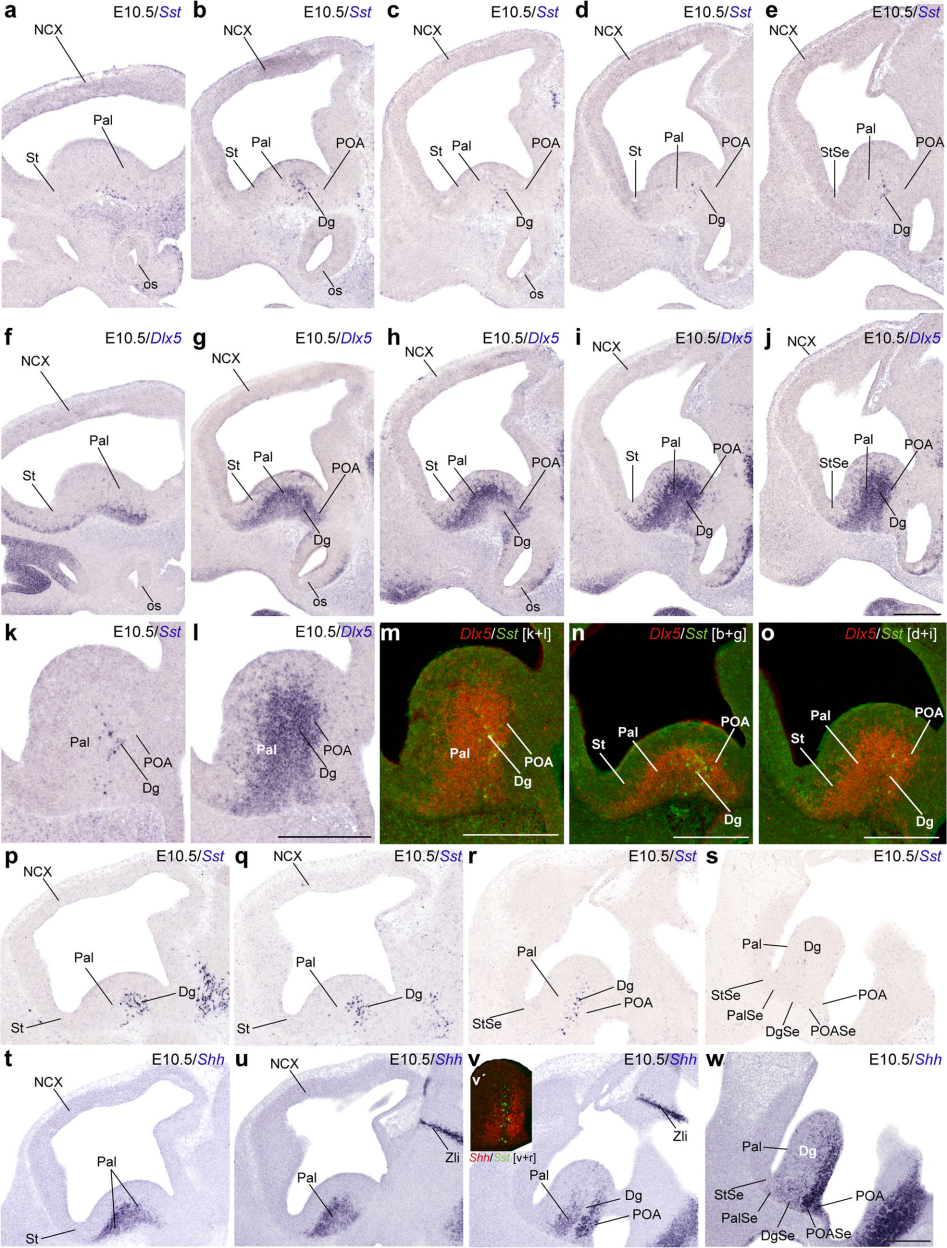
Lateromedial series of sagittal (adjacent) cryostat sections through the MGE at E10.5, illustrating the topography of the earliest *Sst* cells relative to other markers, *Dlx5* and *Shh*: **a**–**e**, **k** *Sst*; **f**–**j**, **l** *Dlx5*; **m**–**o** pseudo-color overlap of *Sst* and *Dlx5* images for the indicated levels; **p**–**s** *Sst* (different specimens); **t**–**w** *Shh*; *inset* to **v** pseudo-color overlap of **r** and **v**. Note the *Sst* cells clearly occupy a restricted domain within the *Dlx5*-positive MGE mantle, which is intercalated between the pallidal and preoptic domains labeled with *Shh* signal

**Fig. 3:**
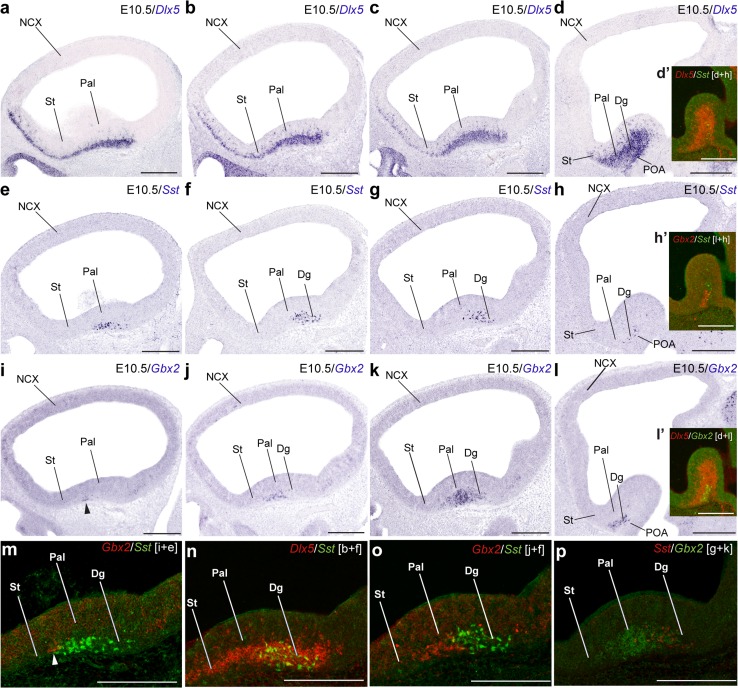
Lateromedial series of sagittal (adjacent) cryostat sections through the MGE at E10.5, illustrating the topography of the earliest *Sst* cells relative to other markers, *Dlx5* and *Gbx2*: **a**–**d** *Dlx5*; **e**–**h** *Sst*; **i**–**l** *Gbx2*; **d**′, **h**′, **l**′, **m**′–**p**′ various pseudocolor overlap comparisons; the markers and levels are indicated. Note lack of overlap between *Sst* cells and the pallidal expression of *Gbx2* (which does overlap with *Dlx5*; see **n**)

**Fig. 4:**
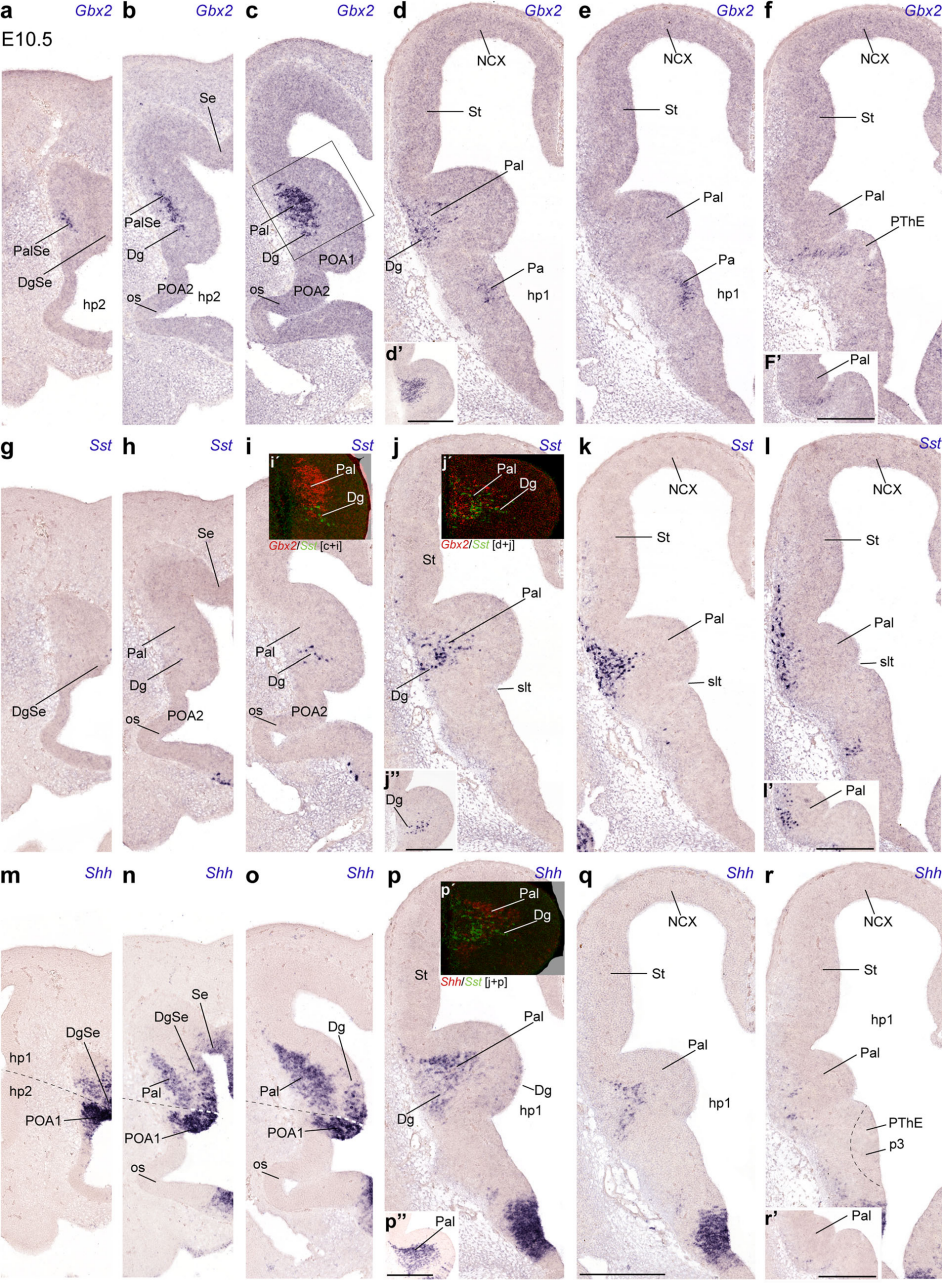
Rostrocaudal series of topologically transversal cryostat sections through the MGE (see plane in Fig. 1a) at E10.5, illustrating in correlative adjacent sections the topography of the earliest *Sst* cells relative to other markers, *Gbx2* and *Shh*: **a**–**f** *Gbx2*; **g**–**l** *Sst*; *inset* **l**′ detail of section caudal to (**l**); **m**–**r** *Shh*; *insets* **i**′, **j**′, **p**′ pseudocolor overlaps of the indicated markers and levels. Note lack of overlap of *Sst* cells with the *Gbx2*- and *Shh*-positive pallidal mantle elements. Pallidal *Gbx2* cells occupy in general more rostral levels than *Sst* cells, and tend to respect the pallidal marginal stratum. Note also the incipient subpial migration of *Sst* cells across the marginal pallidal stratum into the striatum (**j**–**l**). As regards *Shh* expression, the POA1 ventricular zone appears strongly labeled, and clearly produces a migrating cell stream in the mantle that invades selectively the pallidum (**n**, **o**). The adjacent Dg ventricular zone shows less intense and patchy *Shh* signal as well, all the way to the septal end of this band, which diminishes gradientally toward the amygdaloid pole of the MGE, but does not seem to contribute to the preoptic pallidopetal cell stream in the mantle (**m**–**o**)

**Fig. 5:**
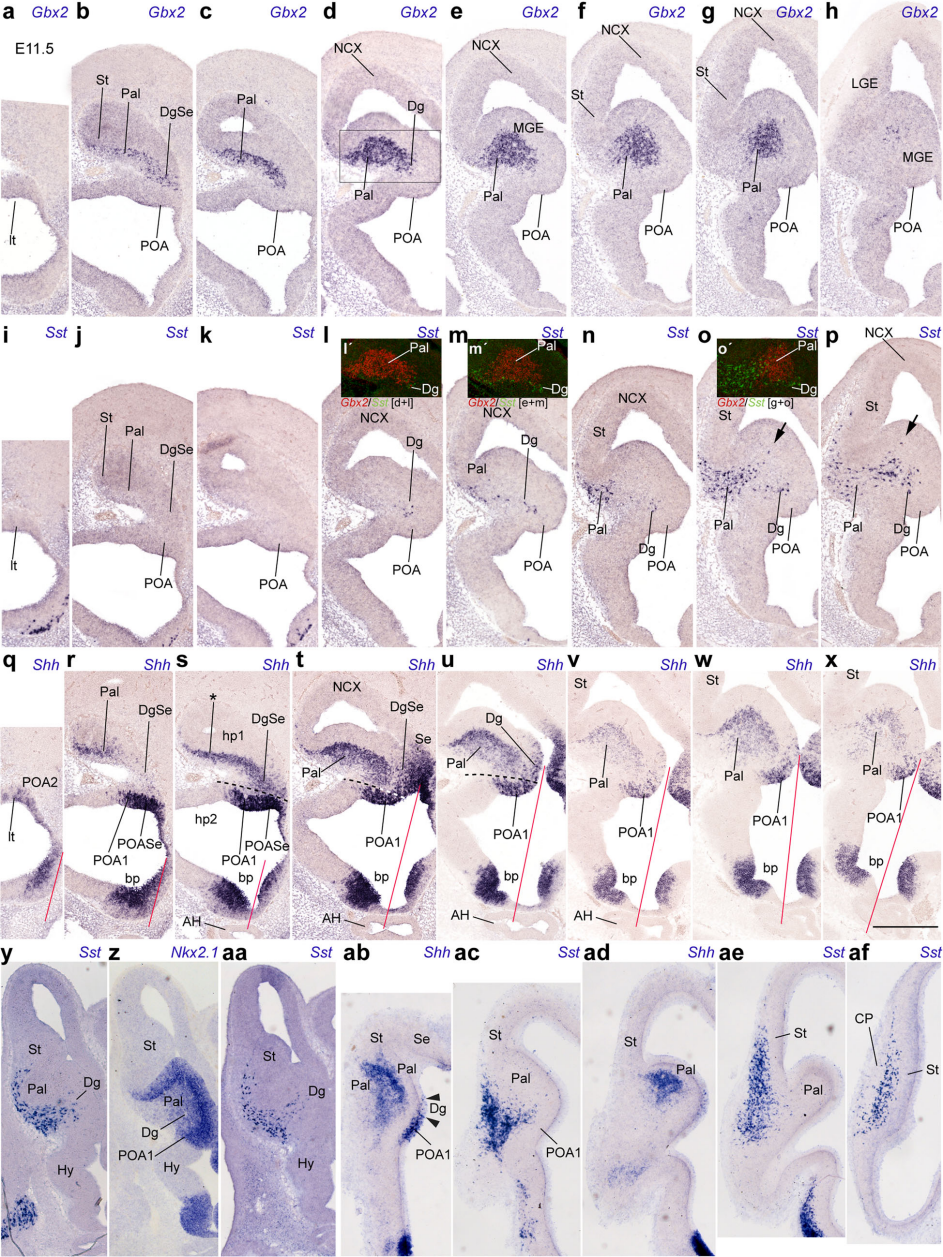
Rostrocaudal series of topologically transversal cryostat sections through the MGE (see plane in Fig. 1a) at E11.5, illustrating in correlative adjacent sections the topography of the *Sst* cells relative to other markers, *Gbx2,* *Shh,* and *Nkx2.1*: **a**–**h** *Gbx2*; **i**–**p** *Sst*; **q**–**x** *Shh*; **y**, **aa**, **ac**, **ae**, **af** *Sst*; **z** *Nkx2.1*; **ab**, **ad** *Shh*; *insets* **l**′, **m**′, **o**′) pseudocolor overlap of the indicated markers and levels. The *red straight lines* entered into panels **q**–**x** indicate the midplane. At this stage, the Gbx2-positive pallidal population extends farther caudalwards, but still essentially does not overlap with the *Sst* cells at the Dg mantle; isolated *Sst* cells that apparently do overlap with Pal are marked with *arrows* in **o**, **p**. Note progression of subpial migratory invasion of the striatum by *Sst* cells (**n**–**p**, **y**, **aa**, **ac**, **ae**, **af**). *Shh* labeling of POA1, Dg, and Pal agrees with the description in Fig. 4; the pallidal *Shh*-positive mantle respects the marginal stratum occupied by migrating Sst cells (**ab**–**ae**)

**Fig. 6:**
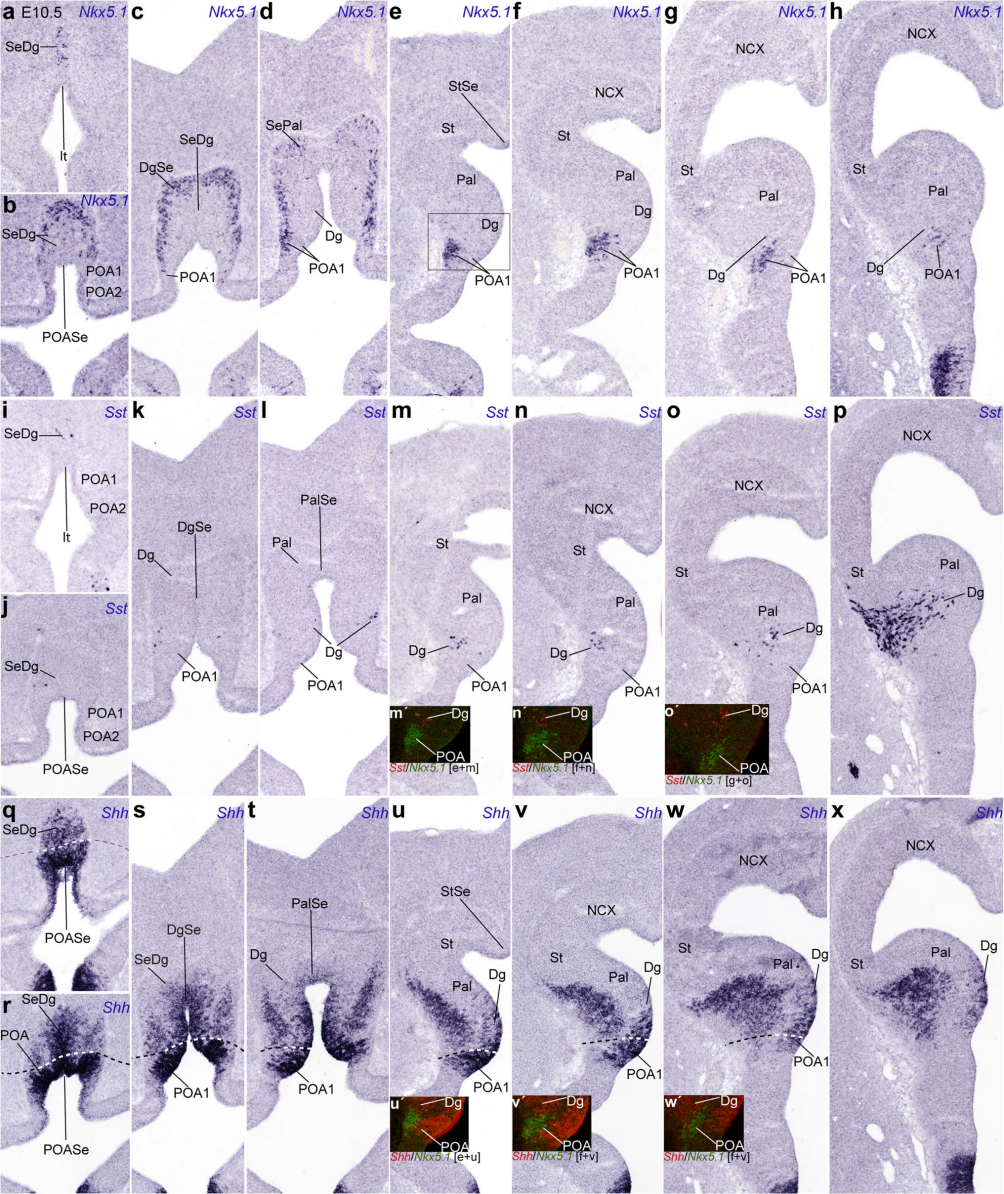
Rostrocaudal series of topologically transversal cryostat sections through the MGE (see plane in Fig. 1a) at E10.5, illustrating in correlative adjacent sections the topography of the *Sst* cells relative to other markers, *Nkx5.1* and *Shh*: **a**–**h** *Nkx5.1*; **i**–**p** *Sst*; **q**–**x** *Shh*; *insets* **m**′–**o**′, **u**′–**w**′ pseudocolor overlap of the indicated markers and levels. There are abundant *Nkx5.1* cells in the preoptic and diagonal-septal neighborhoods (**a**–**d**), as well as in the POA1 mantle layer (**e**–**h**), without significant overlap with *Sst* cells. In **p**, a particularly favourable section plane demonstrates the continuity of *Sst* cells originated selectively at the Dg domain with the incipient migratory phenomenon at the marginal stratum, without apparent implication of the pallidal domain. Note some *Sst* cells are adjacent to the POA1 mantle, without intermixing (*insets* **m**′–**o**′). In contrast preoptic *Nkx5.1* mantle cells are continuous (and partly mixed) with the pallidopetal *Shh*-positive migrating cells in the mantle (*insets* **u**′–**w**′). The ventricular zone of Dg clearly expresses patchily *Shh* (in a septo-amygdaloid decreasing gradient; **s**–**x**)

**Fig. 7:**
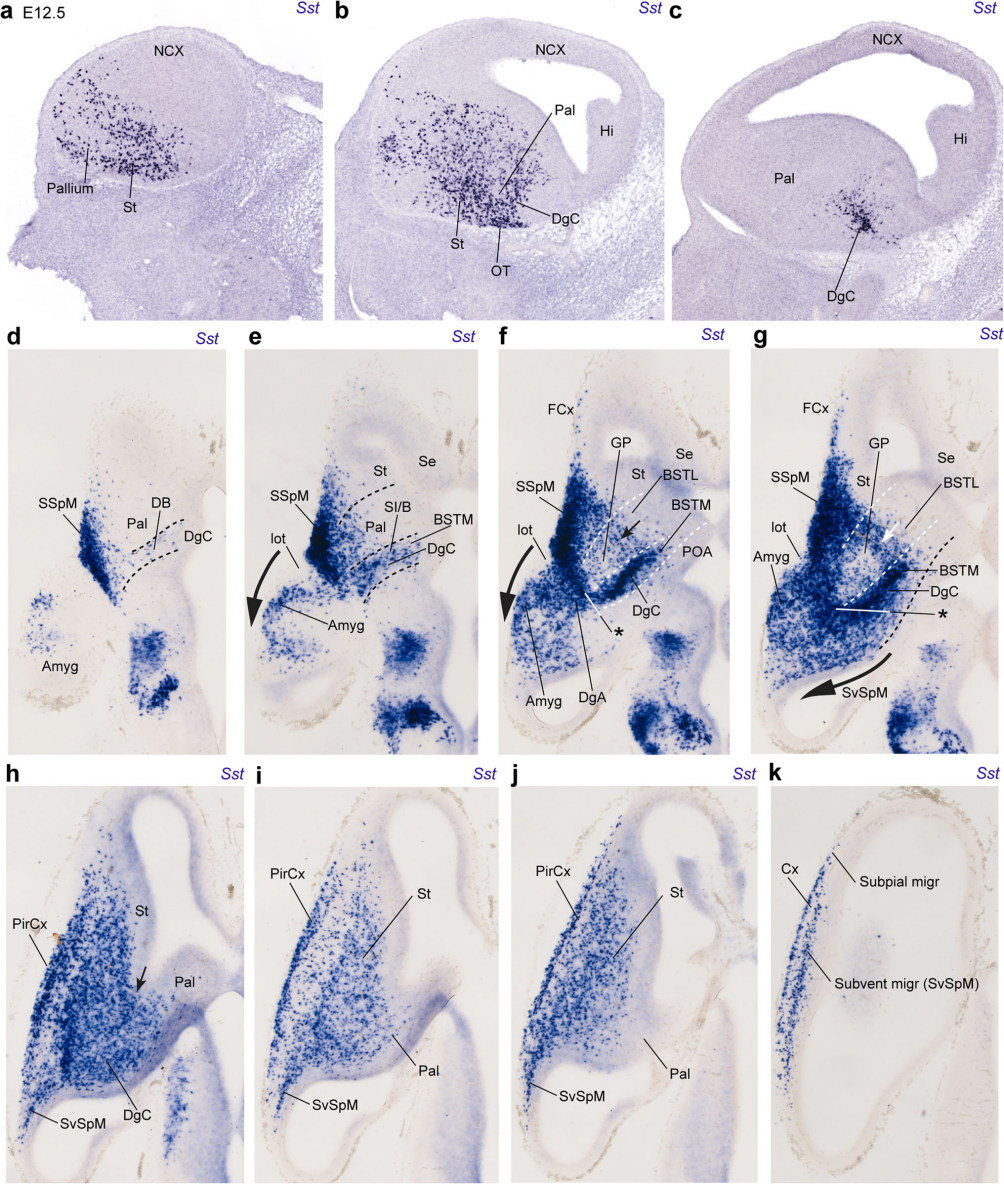
Examples of sagittal and horizontal sections through E12.5 embryonic brains, showing the migratory dispersion of *Sst* cells at this stage. **a**–**c** Lateral to medial set of selected sagittal sections showing the progress of the invasion of the olfactory tuberculum (OT) and striatum (St), as well as the incipient subpial tangential invasion of the pallium (mainly olfactory and insular cortex primordia); the medialmost section **c** shows a restricted topography of *Sst* cells within a central area of the Dg, eschewing the larger pallidal mantle, except at its marginal stratum seen in (**b**). **d**–**k** Ventrodorsal series of horizontal sections, illustrating the septo-amygdaloid dimension of the studied distribution of *Sst* cells; the striatal, pallidal, and diagonal domains are delimited tentatively one from another by oblique *white* or *black dash lines*. The marginal stratum of the whole olfactory tuberculum is occupied by the dense subpial subpallial migratory stream, where *Sst* cells stemming from the Dg domain are seen to arrive (SSpM; Dg; **d**–**g**). Rostrally, labeled cells extend into frontal cortex (FCx); caudally large subpial and subventricular streams of *Sst* cells invade non-homogeneously the pallial amygdala, beyond the DgA region of the subpallial amygdala (large arrows in **e**–**h**). Note as well the existence of *Sst* cells migrating subventricularly across the pallidum into the striatum (small arrows; **f**, **g**). The piriform cortex primordium (largely prospective layer III) appears strongly labeled (PirCx; **h**–**j**)

**Fig. 8:**
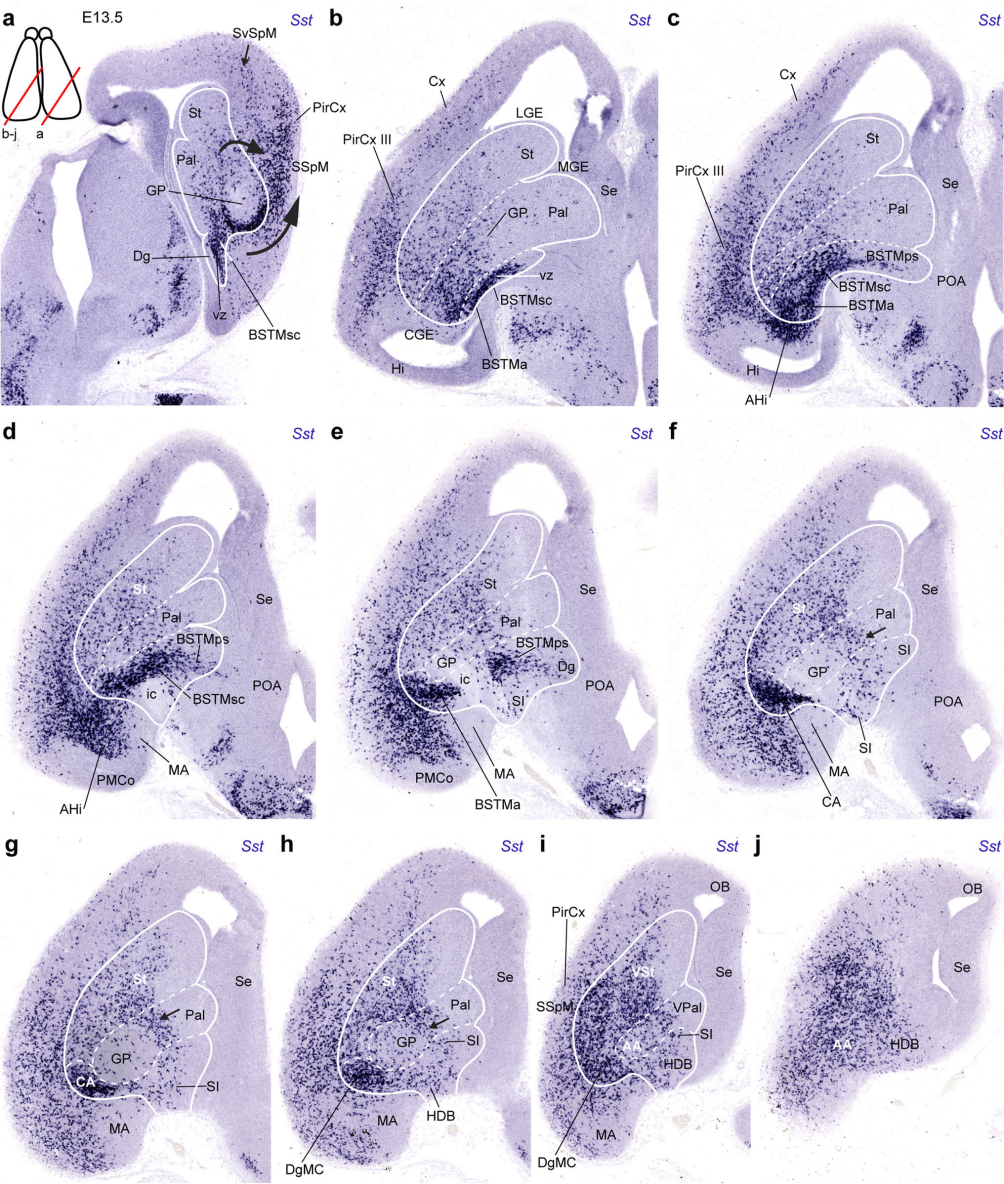
Examples of oblique transversal sections through an E13.5 embryonic brain, showing the migratory dispersion of *Sst* cells at this stage. The plane of section is indicated by *red lines* in the *inset* to **a** (the section in **a** corresponds to the right hemisphere, while the other sections illustrate the opposite side). **a** The obliquity of this section (see *inset*) aligns the strongly labeled Dg source of *Sst* cells (Dg) with the path of their migration deep and superficial to the eschewed globus pallidus (*curved* *arrows* GP), finally converging at the SSpM (at the olfactory tuberculum), and advancing into the olfactory cortex (PirCx), as well as into subpial and subventricular streams targetting cortical pallium (SvSpM). **b**–**j** The oblique septo-amygdaloid section plane obtained in these images (compare *inset* at **a**) is aligned with the diagonal domain, and in general with the three evaginated subpallial domains (delineated as a whole by a *white contour line*, with *dashed internal* *limits*); the series starts caudally close to the lateral ventricle (**b**; see the CGE, LGE, MGE bulges) and progresses into the olfactory tuberculum at **i**, **j**. The maximal density of labeled cells coincides with the Dg ventricular zone (**b**, **c**) and associated periventricular stratum, which forms the supracapsular arch of the medial bed nucleus striae terminalis complex (BSTMsc) over the internal capsule (ic; **d**, **e**); beyond this level, the amygdaloid end of the Dg arch displays a very dense aggregate of *Sst* cells, identified first as the amygdaloid BSTM nucleus (BSTMa) and then as the CA (the primordium of the central amygdaloid nucleus, lateral part) (**e**–**g**). The latter is continuous superficially—close to the SSpM and the OT—with the diagonal magnocellular nucleus (DgMC; **h**, **i**; this was classically misidentified as ‘preoptic magnocellular nucleus’). The other end of the Dg arch constitutes the paraseptal region of the BSTM, which limits with the septum (Se) and the preoptic area (POA) (BSTMps; **c**–**e**; compare Fig. 1 **b**). Ventral to the globus pallidus there are dispersed *Sst* cells within the substantia innominata and the horizontal part of the diagonal band formation (SI; HDB; **e**–**j**). The pallial amygdala shows substantial invasion by *Sst* cells of its amygdalo-hippocampal and basolateral/basomedial areas (AHi; **c**–**g**); in contrast, the medial amygdala (MA) and the posteromedial corticoid area (PMCo) largely remain devoid of these cells (**d**–**i**)

The first telencephalic *Sst* signal was detected in a restricted sector of the subpallial mantle zone from E10.5 onward (Figs. 2, 3, 4, 5, 6, 7, 8; not present at E9.5 and E10); this sector was ascribed to the prospective diagonal area, since it appeared as a thin band intercalated between ampler areas of the incipient MGE that seemed to fall into the pallidal and preoptic domains. To corroborate this analysis, we compared in alternating sagittal sections the topography of *Sst* cells relative to domains expressing either *Dlx5* (Figs. 2f–o,  3a–d), *Shh* (Fig. 2t–w), *Gbx2* (Fig. 3i–l) or *Nkx2.1* (E11; Fig. 5z). *Dlx5* is expressed in all subpallial domains, and certifies the source is subpallial (Bulfone et al. 1993; Eisentat et al. 1999). *Shh* is strongly expressed in the dorsal preoptic ventricular and mantle zones (POA1 of the Allen Developing Mouse Brain Atlas), as well as in cells that migrate selectively from there into the pallidal mantle (Pal) (Gelman et al. 2009). *Gbx2* is selectively expressed in the Pal mantle (Bulfone et al. 1993; Chen et al. 2010; Flandin et al. 2010), and *Nkx2.1* is positive in the pallidal, diagonal (Dg) and preoptic (POA) domains, excluding the striatum (St) (Lazzaro et al. 1991; Shimamura et al. 1995; Sussel et al. 1999; Puelles et al. 2000; Flames et al. 2007; García-López et al. 2008).

The analysis of this material showed that the precocious *Sst* cells lie within the subpallial domain that coexpresses *Dlx5* and *Nkx2.1* (i.e., it excluded the St as a source; Figs. 2a–o,  3a–h, o, 5z). Lateral sagittal sections showed some *Sst* cells aligned subpially, apparently migrating tangentially in a marginal position within the Pal (Fig. 2a, f). More medial sagittal sections contained instead *Sst* cells disposed radially within a narrow intermediate wedge of MGE mantle zone, which ends close to the ventricular zone (i.e., respecting the rostrolateral Pal and the caudomedial POA domains; Fig. 2b–e, g–s). This intercalated domain corresponds within our model to the Dg (Fig. 1b). This interpretation is particularly supported by comparison of these *Sst* cells with the *Shh*-expressing ventricular and mantle zones, which do not include the Dg at these section levels (Fig. 2t–w; see also inset v′).

The scenario is similar in a slightly more advanced E10.5 embryo in which we compared *Sst* with *Dlx5* and *Gbx2* (Fig. 3). The *Sst* population appears in a largely separate domain, the Dg, which is intercalated between the *Gbx2*+ Pal mantle and the *Gbx2*− POA mantle (Fig. 3a–p). The *Sst* cells observed most laterally were again disposed tangentially in the pallidostriatal marginal zone (Fig. 3e), whereas a straightforward radial stream was progressively observed more medially (Fig. 3h).

The spatiotemporal sequence recorded at these initial stages accordingly suggests that precocious *Sst* cells are selectively produced at the Dg domain, wherein they migrate towards the marginal zone. At this stage, most of them seem to advance subpially lateralward, entering the Pal and later the St, always in a marginal position.

### Early telencephalic *Sst* cells in transverse sections

A more precise mapping of the precocious *Sst* cells and their incipient tangential migration relative to the diverse subpallial domains was obtained by examining sections cut transversal to the telencephalic peduncle and the hypothalamus, i.e., the hypothalamo-telencephalic prosomeres (Puelles et al. 2012; see plane T in Fig. 1a). We examined in this way several E10.5 and E11.5 embryos (Figs. 4, 5, 6; S1), in which *Sst* expressing cells were compared in adjacent sections to cells expressing either *Gbx2* or *Shh* (Figs. 4a–r, 5a–x), *Nkx5.1* or *Shh* (Fig. 6), or *Shh* or *Lhx7/8* (Fig. S1).

The rostralmost sections in Fig. 4 intersect the optic stalk, the preoptic area and the septal area (Fig. 4a–c, m–o). Most pallidal *Gbx2* cells lie superficial to the large ventricular/subventricular zone, mainly in the rostral half of the MGE (Pal; Fig. 4c); their number diminishes across the local paraseptal transition (PalSe) into the pallidal septum (SePal; Fig. 4a, b), and also caudalwards (Pal; Fig. 4d). The caudal pole of the Pal domain, which approaches the prospective amygdala, is still devoid of such cells (Pal; Fig. 4e, f, f′). The radially disposed *Sst* neurons of the Dg area are observed just under the maximum of pallidal *Gbx2* cells (Dg; Fig. 4i, i′, j′; the insets Fig. 4d′, j′′, p′′ correspond to a section level intermediate between I and J). Pseudocolored overlap comparison of these two markers (Fig. 4i′) suggested that there are no double-labeled cells, though there are some intermixed units. The *Sst* cells largely are arranged radially underneath the pallidal mass of *Gbx2* cells (compare also Fig. 4d′ and j′′). No *Sst* cells were found at more rostral section levels, where Dg continues into the septum (Fig. 4g, h), irrespective of the proximity of pallidal *Gbx2* cells. In contrast, more caudal sections at levels where pallidal *Gbx2* cells diminish in number displayed a sizeable *Sst* population, which partly appeared intermixed with the caudalmost pallidal *Gbx2* neurons (Fig. 4d, j, j′), and then massively aggregate at the marginal Pal mantle zone, partially invading as well the St domain, always subpially (Fig. 4k, l). Caudally, similar cells are also present at the caudal amygdaloid pole of the MGE or Pal (Fig. 4, inset l′). Interestingly, the section levels where many tangentially migrating *Sst* cells are found are largely devoid of pallidal *Gbx2* cells (Fig. 4).

On the other hand, pallidal *Shh* cells observed in adjacent sections have at this stage a rather rostral topography, agreeing in general with that of *Gbx2* cells, though the *Shh* population is clearly more abundant and extensive rostrocaudally (Fig. 4m–r). As regards expression of *Shh* at the ventricular zone, there is none within the Pal domain of the MGE, whereas a strong signal was found at the part of the preoptic area that builds the medial end of the MGE (Fig. 4m–o); this seems to be the progenitor domain where the cells migrated into the Pal mantle originate. This lateral *Shh*-expressing sector of preoptic neuroepithelium is only present rostrally, consistently with the postulated ascription of the POA to the hypothalamic prosomere 2 (hp2); it limits with the Dg across the hp2/hp1 border (Puelles et al. 2012; Fig. 1).

The Dg ventricular zone shows in contrast weak and patchy expression of *Shh* (Fig. 4m–p), and it is unclear whether it contributes *Shh* cells to the corresponding mantle domain (Dg; Fig. 4p); this includes the transitional paraseptal diagonal area lying between Dg and Se proper (DgSe; Fig. 4m, n). Similar weak ventricular *Shh* expression was found to a limited extent at the median septum (Se; Fig. 4n). On the whole, the subpallial mantle zone contains numerous *Shh* cells, which bridge the distance between the POA/Dg sources and the postmigratory Pal domain, particularly at rostral section levels, where the *Shh* neurons partly overlap with presumably intrinsic pallidal *Gbx2* ones (Fig. 4a–c, m–p); these cells do not penetrate subsequently the striatum (St; Figs. 5, 6). Their number decreases toward the septum, as well as caudalwards, beyond the transverse level where the *Sst* cells first appear (Fig. 4p–r). At these caudal levels, the *Sst* cells migrating through the marginal Pal adopt a position that is largely superficial to the *Shh*+ mantle stratum (Fig. 4j–l, p–r). Curiously, the POA mantle zone does not show radial accumulation of *Shh* cells at all (Fig. 4m–o).

Similar transversal sections obtained at E11.5 show minimal changes (Fig. 5). The pallidal population of *Gbx2* cells is now more extensive, reaching more caudal levels of the MGE (Fig. 5a–g). This caudal prolongation adopts a rounded shape centered in the MGE and does not invade the superficial stratum of the local mantle. *Sst* cells are still absent at the rostralmost levels of the subpallium. They first appear in a radial arrangement close to the Dg ventricular zone at the section levels that contain the maximum of *Gbx2* cells (Fig. 5d–g, l–q; compare also horizontal sections in Fig. 5y, z, aa, which illustrate the restricted topography of postmitotic *Sst* cells relative to the *Nkx2.1*+ MGE complex and the incipient migration into the St). Overlap comparison of these patterns indicates again that the Dg *Sst* cells lie precisely underneath and outside the Pal *Gbx2* cells (insets Fig. 5l′, m′, o′), though some positive cells were observed close to the pallidal subventricular zone (small arrows; Fig. 5o, p). The caudal sections through this area illustrate again numerous *Sst* cells passing into the marginal Pal stratum (circumventing the central mass of *Gbx2* cells), and penetrating tangentially the striatal marginal zone (Fig. 5n–p; see also horizontal sections in Fig. 5ac, ae, af, where pioneering invasion of the pallial cortical plate is visible as well). At these caudal levels, the radial stream of *Sst* cells sorting out of the Dg ventricular zone is still observable (Dg; Fig. 5n–p). It seems that the Dg source of *Sst* cells has expanded backward between E10.5 and E11.5. This pattern is also observable in the *Shh*-positive ventricular and mantle cell populations, which are essentially similar to those described at E10.5, except that the pallidal mantle shows now aggregated *Shh* cells also at the more caudal section levels, and the POA ventricular cell source is also more extensive caudalwards (Fig. 5q–x, z, ab, ad). The preoptic mantle remains devoid of radially aggregated *Shh* cells, but remains itself strongly positive (Fig. 5r–x, ab).

We compared in transverse sections the early distribution of *Sst* and *Shh* cells with cell populations expressing *Nkx5.1* (*Hmx3*) (Fig. 6). This transcription factor was reported to be a selective marker of the preoptic area (Wang et al. 2000; Gelman et al. 2009). At 10.5, there are two quite different expression domains at rostral and caudal section levels, respectively (Fig. 6). In rostral transverse sections behind the septum, there appears a distinct marginal stratum of *Nkx5.1* cells, which seems associated to the transition of the Dg domain into the septum, that is, to the paraseptal DgSe area and the septal SeDg area (Fig. 6a–d). Other more sparsely distributed labeled cells possibly might be ascribed to the septal SePal area (Fig. 6d). Most of these cells lie superficial to the migrating *Shh* cells that emerge from the POA and Dg domains and target the Pal (compare Fig. 6q–t). The rostral part of the POA1 area shows a transition into the septum (via the POASe area), which largely lacks *Nkx5.1* cells (Fig. 6a–c). In contrast, as we proceed into more caudal transverse sections, the paraseptal Dg population disappears and a distinct preoptic *Nkx5.1* population associated to the POA1 mantle zone (at the ventral end of the MGE) becomes apparent; this forms a distinct mantle domain that extends caudalwards with progressively fewer and deeper cells, always found underneath the Dg domain populated by *Sst* cells (POA1; Fig. 6d–h, l–p). The strict association of the patch of *Nkx5.1*+ cells with the *Shh*+ ventricular zone of the POA1 area is demonstrated in the insets Fig. 6u′, v′, w′. Overlap comparison of the *Nkx5.1* elements in the preoptic mantle with *Sst*+ cells in the Dg area illustrates that the latter lie strictly above the *Nkx5.1*+ preoptic ones (Fig. 6d–h, l-p and insets 6m’, n’, o’). In this specimen, we also observed sparse *Sst*+ cells at the DgSe and SeDg areas (Fig. 6i, j, k). These results support that the *Sst* cells are produced independently of the preoptic area, largely caudal to the paraseptal diagonal transition into the septum, and do not invade the preoptic area in their radial and tangential early migrations (see Fig. 6p; note this pattern respects the hp1/hp2 boundary).

Finally, we also compared *Lhx7*-*8* expression in transverse sections with the studied *Sst* and *Shh* patterns. *Lhx7*-*8* is a general marker for Pal, Dg, and POA, thus offering a contrast with the more selective *Sst* and *Shh* signals (Grigoriou et al. 1998; García-López et al. 2008; Zhao et al. 2003). We observed that the pallidal mantle expresses massively *Lhx7*-*8*, occupying even the marginal stratum that is devoid of *Shh* cells (Figs. S1m–r). There are also many marked cells scattered in the subventricular zone of the Pal domain, particularly at rostral section levels, where these elements are relatively more numerous in the medial part of the MGE than laterally, and the corresponding medial mantle stratum is also more massively populated (Fig. S1m–o). The Dg domain, as defined by the weak and patchy ventricular expression of *Shh*, seems to contribute likewise to this mz/vz *Lhx7*-*8* pattern, though it shows a thinner positive mantle (Fig. S1o–q). At the diagonal part of the septum (median SeDg area), only a positive mantle zone was present (Fig. S1m; compare with the neighbouring pallidal part of the septum –SePal- see the inset S1m′), suggesting a possible tangential migration from more lateral origins. Once the POASe area is reached in the series of sections, the *Lhx7/8*+ mantle zone disappears (Fig. S1n). At a single section level, there was a marginal line of labeled cells at the POA2 (*Shh*-negative) area (Fig. S1o). More caudally, there appear instead *Lhx7/8* cells in the POA1 mantle (ventral MGE), which is distinctly thinner than that present at the Pal/Dg complex (Fig. S1p-r). The *Lhx7*-*8*+ Pal mantle population diminishes in cell density caudalwards, coinciding with the place where *Sst* cells course marginally through the Pal into the St (Fig. S1e, f, q, r). Though there is some topographic overlap between *Sst* and *Lhx7/8* cells at the Dg domain and neighbouring Pal, many tangentially migrating *Sst* cells clearly do not express *Lhx7/8* (compare Fig. S1d–f with S1p–r). It is unclear whether this implies that these cells downregulate an initial postmitotic expression of *Lhx7*-*8*.

### Progress of telencephalic *Sst* cell populations between E12.5 and E14.5

Migrating *Sst* cells streaming tangentially through the subpial part of the central subpallium start to invade the striatum and the pallium at E11.5 (Fig. 5y, aa, ad, ae, af). At E12.5, sagittal sections illustrate the arrival of many migrating *Sst* cells at the rostrolateral part of the telencephalic pallium either via the massive superficial subpallial migratory stream (Fig. 7a, b), or via the less populated subventricular striatal stratum (Fig. 7k), best observed in horizontal sections (small arrows; Fig. 7f–k). In contrast, medial parts of the pallium and the paraseptal and septal parts of the subpallium are devoid of *Sst* cells (Fig. 7c). The horizontal sections clearly show the spatial relationship between the SSpM and the presumed origin of the *Sst* cells, the central diagonal area territory (DgC); the latter appears disposed as an oblique (diagonal) band of labeled cells at the back of the SSpM. The labeled diagonal population is sparse superficially at the site of the prospective diagonal band nuclei (DgC; Fig. 7d), but increases significantly at the corresponding intermediate and periventricular strata (DgC; Fig. 7e–g). In addition, less abundant *Sst* cells apparently also course rostrolaterally at various depths through the central pallidal and striatal territories, finally incorporating into the SSpM or the subventricular pallial zone, or penetrating extensively the central striatal mantle (Fig. 7e–j). There are clearcut caudolateral and rostromedial boundaries of the SSpM, which possibly coincide with the limits of the central part of the subpallium versus the amygdaloid and paraseptal/septal sectors (Fig. 1b). The amygdaloid area (consisting of both subpallial and pallial parts) is also incipiently invaded by *Sst* cells that either stream back tangentially from the DgC domain, or originate locally from the amygdaloid sector of the Dg domain (DgA; asterisks in Fig. 7f, g). *Sst* cells invade the pallial amygdala coursing either superficially or periventricularly (large arrows in Fig. 7e–g; see also 7h–j). Interestingly, the incipient globus pallidus developing within the central pallidal mantle seems to be relatively non-permissive for the reported diagonal central migration into the striatum and the cortex, so that it tends to be eschewed by the superficial and deep migrating *Sst* cells, and thus appears as a nearly unlabeled cell mass adjacent to the DgC band (GP; Fig. 7f, g). Migrating cells reach the SSpM passing all around the GP (Fig. 7e–h). The cell stream connecting the caudal DgC and the DgA with the neighbouring SSpM is particularly dense (Fig. 7e–g); data will be shown below suggesting that this locus relates to the prospective *Sst*-positive part of the central amygdaloid nucleus. *Sst* cells are most dense subpially at the primordium of the olfactory tuberculum, whose superficial corticoid layer is not yet distinguished at E12.5 (TO; Fig. 7d–g). *Sst* cells moving past the striatum clearly invade the primordium of the prepiriform cortex, before reaching the neocortical marginal layer (PirCx, Cx; Fig. 7h–k); fewer cells enter subpially the rostrolateral frontal pallium via independent subpial and subventricular routes (Fig. 7a, b, f–k).

**Fig. 9:**
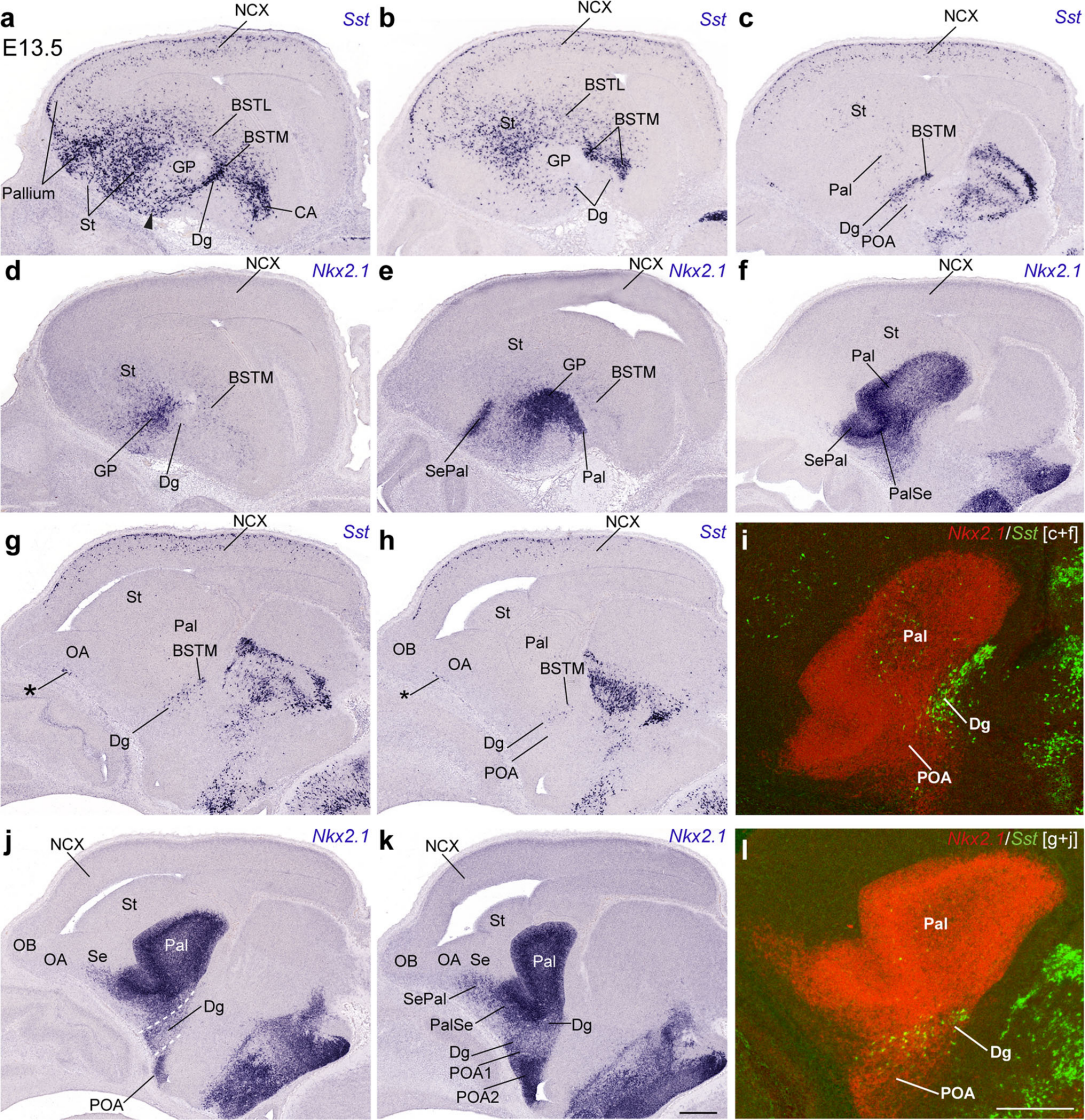
Examples of sagittal sections through an E13.5 embryonic brain, showing more advanced migratory dispersion of *Sst* cells (**a**–**c**, **g**, **h**), and the relationship of the Dg radial domain with the domain of expression of *Nkx2.1* (**d**–**f**, **j**, **k**); **i**, **l** pseudocolor overlap of both markers at the indicated levels. Note that at this stage, the Dg ventricular zone and at least part of its periventricular BSTM formation fall outside the domain of expression of *Nkx2.1*. Many *Sst* cells have invaded the isocortical plate at superficial and deep strata

At E13.5 the pioneering *Sst* cells reach the convexity of the cortical mantle, where they appear mainly dispersed among the cortical plate and subplate cells; the developing cingular and hippocampal cortical areas, as well as the medial amygdala, are devoid of labeled cells (Figs. 8a–j, 9a–c, g, h). At this stage, the earlier subventricular migratory stream entering the cortex has largely disappeared, though some dispersed *Sst* cells are still visible within this stratum (arrow in Fig. 8a). The major tangential migratory course is represented by the SSpM observed at the pial surface of the subpallium (not shown); from there *Sst* cells proceed into the pallial mantle lying under the olfactory cortex (SSpM; PirCx; Fig. 8a). The majority of labeled cells approaching the SSpM course behind the globus pallidus, bypassing laterally the internal capsule, whereas fewer cells apparently join this stream passing through the deep corridor passing across the pallidal and striatal mantle (Fig. 8a). In Fig. 8, we enclosed with a black line the areas we estimated to be subpallial, to aid the description of *Sst* cell populations with pallial versus subpallial topographies. The densest pallial *Sst* cells were found at the prepiriform/piriform cortex (mainly layer III; Figs. 8a–h, 9a) and in the pallial amygdala (mainly amygdalohippocampal area; AHi; Figs. 8c, d, 9a). The olfactory bulb primordium is devoid of *Sst* cells (Figs. 8i, j, 9j, k).

**Fig. 10:**
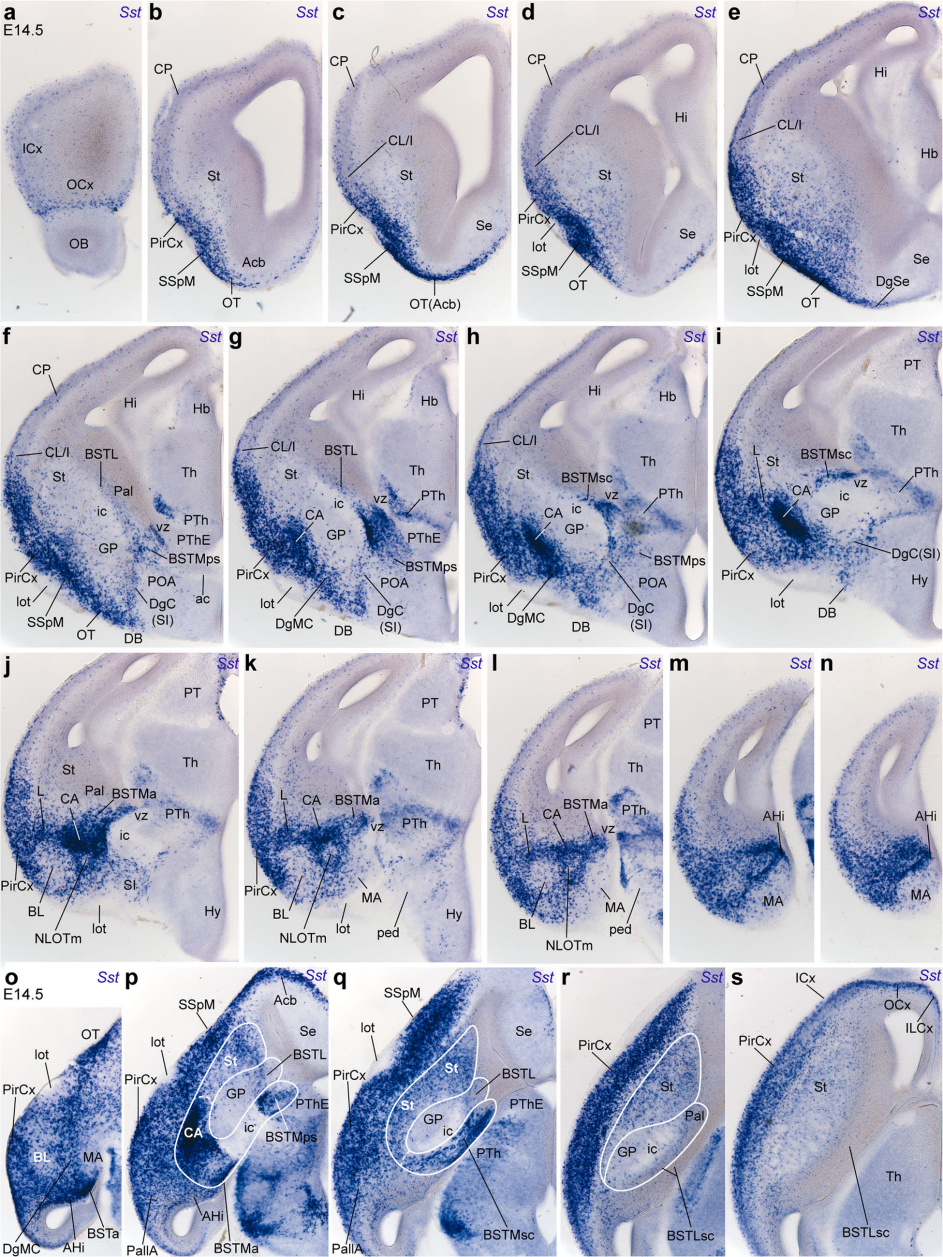
Examples of transversal (**a**–**n**) and horizontal (**o**–**s**) sections through E14.5 embryonic brains, showing the migratory dispersion and increasing differentiative stabilization of *Sst* cells at this stage. **a**–**n** The rostral sections clearly display the superficial subpallial migratory stream advancing from the olfactory tuberculum, just deep to the lateral olfactory tract into the deep stratum of the olfactory cortex and then largely subpially into the insula and isocortical plate (SSpM; lot; CL/I; CP; **a**–**f**); some cells enter medially the septum (Se). The striatal primordium still shows more cells superficially than next to the internal capsule (scarce *Sst* cells at subventricular levels). The pallidum is practically devoid of labeled cells at the globus pallidus, but shows some small elements within its periventricular (supracapsular) BSTL nucleus (GP; BSTL; **f**–**i**). As regards the Dg domain, the periventricular BSTM arch is cut obliquely in this plane of section; we see first the paraseptal component, which approaches the crossing of the anterior commissure and the preoptic area (BSTMps; so-called ‘anterior’ BST; **f**–**h**); the caudal end, composed by the amygdaloid lateral CA and DgMC nuclei, appears close by, internally to the position of the lateral olfactory tract (CA, DgMC; **g**, **h**); both elements are soon united by the intermediate supracapsular portion of the BSTM complex (BSTMsc; **h**–**j**), at shortly thereafter we also see the labeled amygdaloid BST nucleus (BSTMa; **j**–**l**); the radial continuity of the Dg ventricular zone with its pial surface is populated throughout by *Sst* cells dispersed in the substantia innominata, converging at the brain surface upon the diagonal band nucleei (vz; SI; DB; **f**–**l**). Caudolaterally to the CA there appears the pallial amygdala, including the strongly labeled lateral nucleus and the poorly labeled basolateral nucleus (L, BL; **i**–**l**) and the strongly labeled amygdalo-hippocampal area (AHi; **m**, **n**). The medial amygdala (MA) only contains scattered labeled cells. **o**–**s** These horizontal sections offer a complementary view of the same anatomic details distinguished in transversal sections

The central diagonal histogenetic domain (DgC) shows its characteristic oblique band of dense *Sst* cells placed ventromedial to the globus pallidus (central pallidal subdomain) and dorsolateral to the preoptic area (both largely *Sst*-negative; DgC, GP; POA; Fig. 8b–e). The periventricular part of this band arches over the internal capsule, consistently with the subsequent position of the supracapsular BST (bed nucleus of the stria terminalis) complex. The observed dense aggregates of *Sst* cells clearly represent the medial (diagonal) part of the supracapsular BST (BSTMsc), since the lateral BST counterpart belongs to the periventricular pallidum (BSTMsc; Fig. 8a–d; see overall map in Figs. 1b, 10q, r, 12c, d show the distinction between *Sst*-positive BSTM and *Sst*-negative BSTL at E14.5 and E15.5, respectively). Ventral to the level where the internal capsule penetrates the subpallium (closer to the olfactory tuberculum), the caudal end of the supracapsular BSTM arch extends into the amygdala, where we observe the dense radially migrated *Sst* cells of the amygdaloid BST complex (BSTA) and the associated diagonal part of the central amygdala (CA), as well as other *Sst* cells that migrated tangentially into the pallial amygdala, invading mainly the prospective basal and cortical region, and the amygdalo-hippocampal area, but eschewing the medial amygdala (AHi; Fig. 8e–g). On the other hand, the rostral end of the supracapsular BSTM arch reaches rostromedially the paraseptal part of the diagonal BSTM complex (BSTPs); some labeled cells apparently disperse from here into the neighbouring preoptic area (BSTMps, POA; Fig. 8c–f). The intermediate stratum of DgC found underneath the anterior commissure builds the substantia innominata; it only contains sparse *Sst* cells (SI; Fig. 8f, g); such cells become slightly more abundant at the corresponding superficial DgC stratum, occupied by the horizontal nucleus of the diagonal band (HDB; Fig. 8h–j).

At E13.5, the striatal mantle shows a dispersed population of labeled cells, whose density markedly increases near the pial surface (future ventral striatum), and reaches a maximum at the SSpM stream, which lies now deep to the incipient olfactory tuberculum (SSpM; Fig. 8i, j; compare 8a). Coronal sections indicate that this dense subpial stratum is also present, but thinner, at the nucleus accumbens (paraseptal striatum) and the neighbouring striatal septum (not shown). Comparison in adjacent sagittal sections of the expression patterns of *Sst* and *Nkx2.1* (a general marker at early stages of Pal, Dg and POA) illustrates at E13.5 that *Sst* cells are practically absent in the globus pallidus (GP; Fig. 9a, b, d, e), and only the ventral pallidum and the periventricular pallidal around the GP contain some *Sst* cells, which presumably are passing through into the striatum and pallium (Fig. 9c–l). In contrast, the Dg periventricular zone appears characterized by dense *Sst* cells, which form the BSTM primordium (BSTM; Fig. 9c, g, h); this locus is characterized by rather weak, or absent, *Nkx2.1* expression at E13.5 (Fig. 9f, j, k; see also pseudocolor overlaps in i, l). Also the corresponding paraseptal and septal diagonal ventricular zone showed distinctly less *Nkx2.1* signal than the neighbouring paraseptal and septal pallidal and preoptic domains (Fig. 9j–l). This change in molecular background distinctly separates a pallidal *Nkx2.1*+*/Sst*− domain (with strong *Nkx2.1*) from the *Nkx2.1*+*/Sst*+ diagonal domain (with weak *Nkx2.1*).

**Fig. 11:**
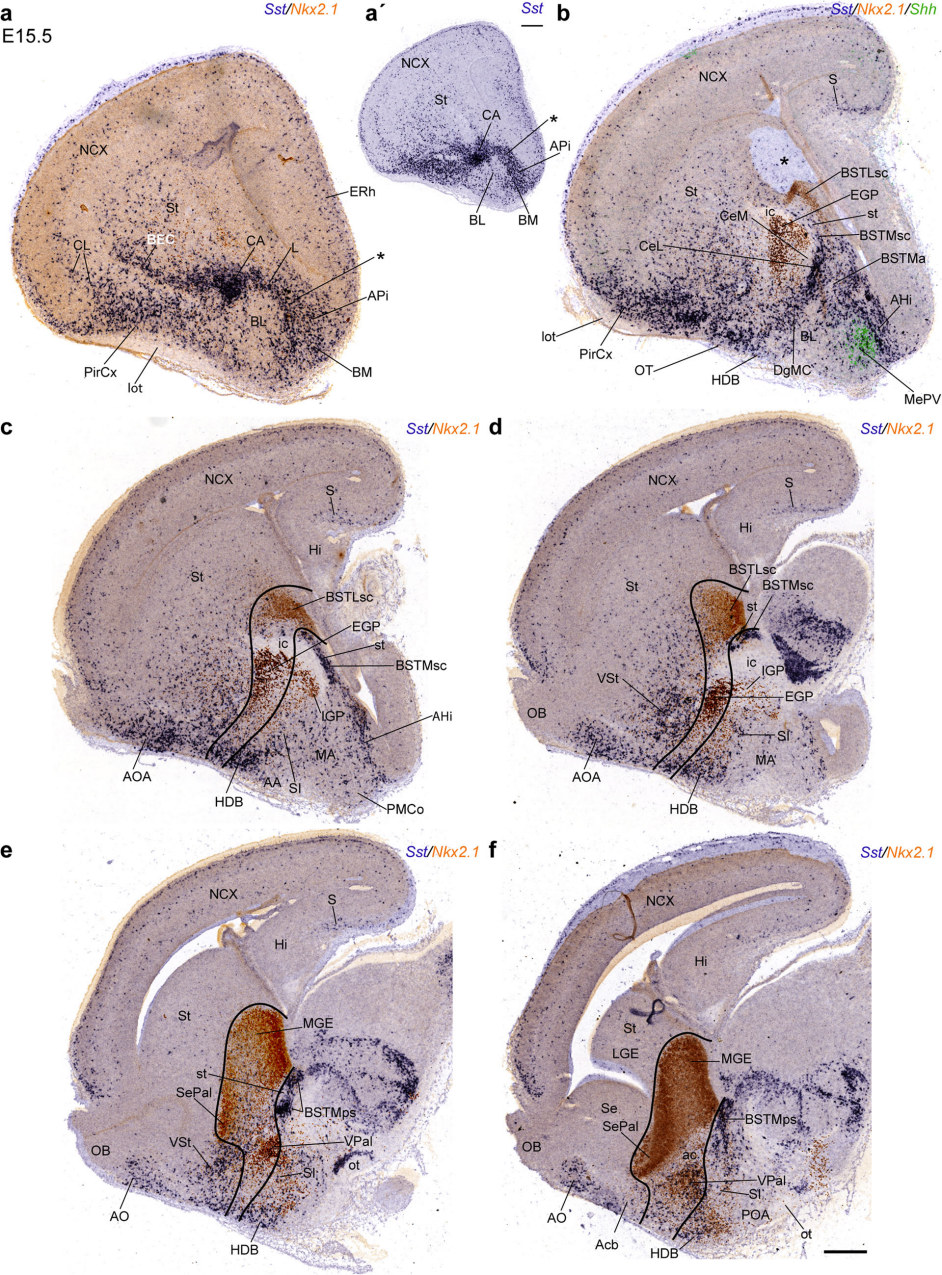
Digitally superposed images of adjacent sagittal sections at 6 section levels through an E15.5 embryonic brain, showing the distribution of *Sst* cells relative to the NKX2.1-immunoreactive domain (**inset a**′ shows a section lying lateral to **a**, reacted only for *Sst* ISH). (**a, a**′) Lateral sections pass through the pallial amygdala, laterally to the globus pallidus; the central lateral amygdalar nucleus is densely labeled (CA; prospective CeL); note sparse *Sst* cells within the neighboring basolateral amygdalar nucleus (BL) and more abundant cells at the lateral and basomedial amygdalar nuclear primordia (L, BM); migrating *Sst* cells have reached the entorhinal cortex, but not yet the hippocampus (ERh; Hi; in **b**, **d**). Labeled cells also aggregate more rostrally within the bed nucleus of the external capsule (BEC). **b**–**d** Section **b** shows, superposed in *green*, the image of *Shh* expression within the medial posteroventral amygdaloid nucleus, a site with scarce *Sst* cells. These more medial sections also intersect the striatum (dispersed labeled cells in a ventrodorsal gradient); next to it appears the globus pallidus (GP) and the supracapsular lateral BST nucleus (BSTLsc), both of which are NKX2.1 immunoreactive and poor in *Sst* cells. The pallidal radial domain ending superficially at the olfactory tuberculum (with many labeled cells), and limiting at the ventricular zone with the *Sst*-positive Dg domain, is tentatively enclosed by *black lines* in **c**, **d**. These *lines* suggest that the NKX2.1-immunoreactive GP is divided topographically into an external (pallidal) portion and an internal (diagonal) counterpart (EGP, IGP; **b**–**d**). The diagonal complex (Dg) includes the densely labeled periventricular BSTMsc formation, the IGP, the NKX2.1-immunoreactive substantia innominata (with relatively few intermixed *Sst* cells) and the superficial horizontal nucleus of the diagonal band (HDB; this has as many labeled cells as the olfactory tuberculum and also contains some NKX2.1-immunoreactive cells). **e**, **f** At these more medial section levels, we reach the medial end of the supracapsular BSTM arch (BSTMsc), moving into its paraseptal portion (BSTMps), next to the anterior commissure (ac) and the preoptic area (POA)

One day later, at E14.5, coronal and horizontal sections illustrate various more advanced aspects of the static and migrating *Sst* populations. Whereas *Sst* cells do not yet enter the olfactory bulb or the anterior olfactory area, the tangentially migrating cells now incipiently colonize the insular, frontal, orbital, infralimbic and anterior cingulate cortical areas (Fig. 10a, s), as well as most of the striatal mantle, the latter in a decreasing gradient toward the ventricular zone (St; Fig. 10b–j, p–s). The piriform cortex primordium lying deep to the lateral olfactory tract appears densely penetrated by *Sst* cells, a differential characteristic with respect to the overlying, less populated claustro-insular complex and other parts of the cortex (PirCx, lot; Fig. 10b–n, o–s); the intervening clearcut boundary separates the newly postulated ventropallial and lateropallial derivatives (see Puelles 2014). Just medially to the lateral olfactory tract, the subpallial subpial stratum is still full of *Sst* cells, which correspond to the SSpM stream traversing the prospective olfactory tuberculum (SSpM; Fig. 10b–f, o–q). These subpial *Sst* cells also extend medialward into the subpallial paraseptal areas next to the septum, but only across the accumbens area (paraseptal St) and the diagonal-septal area (DgSe or paraseptal Dg); interestingly, this does not occur at the intervening pallido-septal area (PalSe or paraseptal Pal) (Acb; PalSe; DgSe; Fig. 10b–e; p). Immediately caudomedial to the olfactory tuberculum, the number of *Sst* cells present at the horizontal part of the diagonal band has increased, but remains less abundant than at the olfactory tuberculum, suggesting absence of a tangential migratory route at this locus (DB; Fig. 10f–h). The DB formation is radially continuous with deeper labeled cells that form a sublenticular population medially and caudally to the conspicuously negative globus pallidus; this intermediate stratum of the central diagonal area corresponds to the classic substantia innominata (DgC/SI; GP; Fig. 10f–i). The corresponding periventricular stratum contains the diagonal supracapsular BSTM (BSTMsc), continuous rostromedially with the corresponding paraseptal sector (BSTMps). The latter’s labeled cells contrast with the completely unlabeled prethalamic eminence behind it (BSTMps; PThE; Fig. 10f–h, p, q). The supracapsular BSTM can be followed caudolaterally over the internal capsule, next to the negative GP (BSTMsc; Fig. 10h, i, q), until it reaches its amygdaloid end (BSTMa; Fig. 10j–l, p). The dense population of *Sst* cells in the BSTMps, BSTMsc, and BSTMa contrasts sharply with the absence of such cells in the adjacent pallidal part of the BST, identified by us as BSTL (BSTL; Fig. 10r, s; see also Fig. 11 and supplementary Fig. S2a, b). Irrespective of its low *Nkx2.1* expression level, the BSTM clearly lies within the *Dlx5*-expressing subpallium (at its border), whereas the neighboring supracapsular migration stream that vehiculates hypothalamic *Otp*-positive neurons into the amygdala passes just medial to the BSTM, outside the *Dlx5*-positive subpallium, within a thin periventricular *pallial corridor* that connects the pallial amygdala with the peduncular hypothalamus (Fig. 3a–c; García-Moreno et al. 2010; Morales-Delgado et al. 2011; Puelles et al. 2012).

The BSTMa in its turn connects ventrolaterally with the densest part at this stage of the diagonal radial migration stream, which approaches superficially the caudal end of the SSpM, passing behind the GP; this very dense radial stream of labeled cells is first found superficially, lateral to the GP, and then laterally to the internal capsule, behind the GP. According to observations at later stages (see below), many of these cells form definitive nuclear derivatives, irrespective that others may enter the SSpM and continue tangentially into the cortex or the striatum. These local radial mantle derivatives of the amygdaloid part of the diagonal area correspond to the primordium of the lateral central amygdala, found next to the BSTMa (CA; Figs. 8f, g, 10g–l, p), and, more superficially, to the primordium of the classic magnocellular preoptic nucleus. The latter term is a clear misnomer (since the locus is well outside the preoptic area, and the POA does not produce *Sst* cells), which leads us to propose renaming it the *magnocellular diagonal nucleus*, or DgMC (this agrees with its topography close –but deep- to the horizontal DB nucleus; DgMC; Figs. 8h, i, 10g–h, o). At section levels caudal to the GP, the dense CA primordium contains an unlabeled anteroposterior stream of cells in its interior, pointing toward the anterior amygdalar area, which we believe corresponds to the migratory stream of the nucleus of the lateral olfactory tract (NLOTm; Fig. 10j, k; Remedios et al. 2004).

At E14.5 the pallial amygdala lying lateral and caudal to the CA displays ventrally a large ovoid area with sparse *Sst* cells, which we believe is the primordium of the basolateral amygdaloid nucleus, and dorsally to it, deep to the PirCx, there appears a cap-like area full of *Sst* cells, which corresponds to the prospective lateral amygdaloid nucleus, as indicated by data at subsequent stages (BL; L; Fig. 10j–l, o–q). The medial amygdala instead shows few labeled cells (MA; Fig. 10j–n, o), whereas the area where the prospective basomedial and corticoid nuclei form contains a moderate amount of *Sst* cells (untagged; Fig. 10k–n, o).

### Progress of telencephalic Sst cell populations between E15.5 and E16.5

**Fig. 12:**
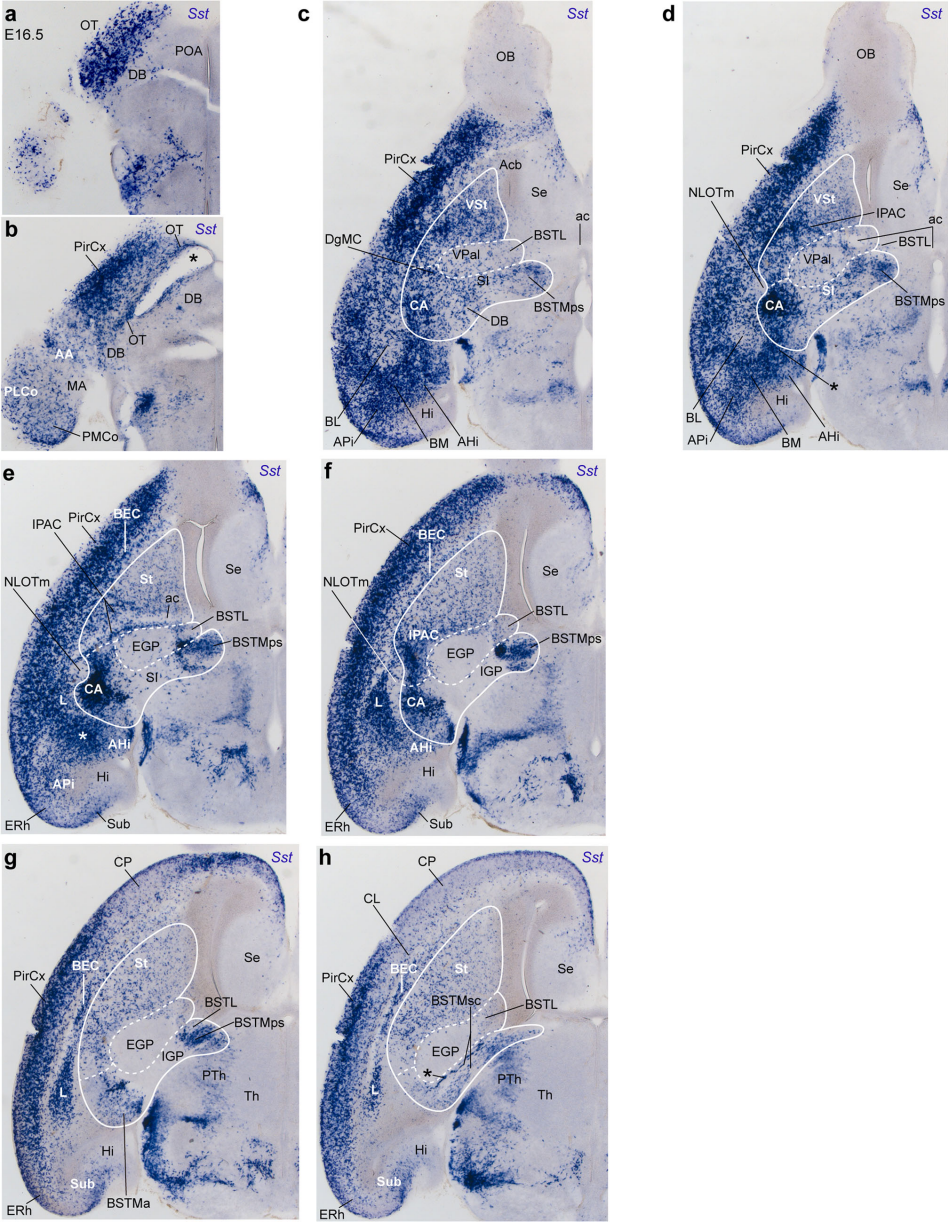
Selected horizontal sections through an E16.5 embryonic brain, showing the telencephalic distribution of *Sst* cells at this stage. The subpallial complex—St, Pal, Dg—is contoured by a continuous white line, and white dash lines separate these three domains. **a**, **b** The olfactory tuberculum (with many *Sst* cells) and the basalmost part of the amygdala are cut tangentially (the latter with labeling of the anterior amygdala, AA, and the posterolateral and posteromedial cortical nuclei, PLCo, PMCo). The *diagonal band* is partly seen (the *asterisk* marks an artefactual distortion). **c**, **d** Levels of section through the crossing of the anterior commissure (ac): intense labeling of the piriform cortex (prospective layer III) continues rostrally into the posterior part of the anterior olfactory area; few cells reach the olfactory bulb, and septal labeling is limited to medial portions (Se). The ventral striatal mantle is well populated by *Sst* cells (VSt), contrasting with the sparser population within the ventral pallidum (VPal). The diagonal domain is represented by the diagonal band (DB), the substantia innominata (SI), and the paraseptal BSTM nucleus (BSTMps). The subpallial amygdala displays the strongly labeled DgMC and CA derivatives, rostromedially to the relatively unlabeled BL nucleus, whereas the amygdalopiriform area (APi), BM, and AHi amygdalar areas are strongly labeled. **e**, **f** The temporal fibers of the anterior commissure traverse the positive IPAC formation (interstitial nucleus of the posterior limb of the anterior commissure) at the back of the striatal mantle, which is limited laterally by a dense population at the bed nucleus of the external capsule, a ventral pallium derivative, jointly with the piirform cortex (BEC; PirCx); caudally, BEC seems continuous with the larger lateral amygdalar nucleus (L), which is also densely labeled. At the interface between L and CA there appears a round or elongated comet-shaped domain devoid of *Sst* cells that corresponds to the migrating primordium of the nucleus of the lateral olfactory tract (NLOTm). At the rostromedial end of the supracapsular BSTM arch there appears the Dg paraseptal BSTM derivative (BSTMps), which now shows several subdivisions. **g**, **h** At these dorsal levels the l amygdalar nucleus diminishes in size, still showing continuity with the BEC, deep to the piriform cortex (PirCx) and next to the striatum (St). A *linear* claustral aggregate can be distinguished in **h** (CL). The amygdaloid and paraseptal ends of the BSTM arch meet over the internal capsule and the IGP, forming the supracapsular region (BSTMsc)

We also analyzed the expression of *Sst* in sagittal sections at E15.5 (Fig. 11) and horizontal sections at E16.5 (Fig. 12). In general, we noted a progressively wider dispersion of *Sst* cells within the pallium, notably including incipient invasion of caudomedial areas such as entorhinal cortex, subiculum, and hippocampus (ERh; S; Hi; Figs. 11a–f, 12c–h). *Sst* cells appear broadly dispersed in all regions of the neocortex (Figs. 11a–f; 12f–h), though there remains a distinctly larger population in the ventral anterior olfactory area, the olfactory tuberculum and the prepiriform and piriform areas (AOA, OT, PirCx; Figs. 11a–f, 12a–h); such olfactory cortex cells subsequently largely populate the adult layer III (see supplementary Figs. S3a, b), suggesting that at the observed intermediate developmental stages there is still a relatively immature state of these allocortical areas. There are scarce labeled cells in the pallial subventricular zone, whereas the marginal stratum contains many cells (Figs. 11a–f, 12e–h). At section levels through the insular cortex, there appears a line of deep aggregated *Sst* cells that are aligned with the lateropallial claustrum (CL; Fig. 12h; see Puelles 2014). This claustral aggregate is separated by label-free white matter from a parallel line of densely labeled cells next to the striatum. The latter cells seem to have invaded deep nuclear derivatives of the ventral pallium that are associated to the external capsule, namely the recently identified bed nucleus of the external capsule (BEC; Puelles 2014), previously known as ‘reservoir’ (Bayer and Altman 1991). The BEC seems continuous caudally with the densely *Sst*-labeled primordium of the lateral amygdaloid nucleus (LA), a larger triangular pallial aggregate also held to derive from the ventral pallium (Medina et al. 2004). The continuity of these two formations is partly interrupted by passing fibers of the posterior limb of the anterior commissure (CL, BEC; Fig. 11a; CL, BEC, LA, ac; Fig. 12e–h). Ventral to the LA, the basolateral amygdaloid nucleus retains its original ovoid aspect and sparse population of *Sst* cells, particularly laterally (BL; Figs. 11a, a′, 12c, d). This primordium is surrounded dorsally and caudally by a relatively dense migratory stream of labeled cells apparently spreading out of the BSTMa (asterisk in Figs. 11a, a′, 12d, e). This intraamygdaloid stream surrounds the BL and connects rostrally and dorsolaterally with the base of the LA (see L; Figs. 11a, 12e–g), and caudoventrally with the amygdalopiriform area and the basomedial nucleus (APi, BM; Figs. 11a, a′, 12c–e). Cells from this stream also extend medialwards into the amygdalohippocampal area, possibly connecting there with a separate periventricular migratory stream (BM, AHi; Figs. 11a, a′, b, c, 12c, d). In contrast with these at least transiently well-populated areas of the pallial amygdala, the anterior amygdala, and the medial amygdalar nuclei, including the *Shh*-positive posteroventral medial nucleus (green in Fig. 11b), show only few dispersed *Sst* cells (AA, MePV, MA; Figs. 11b–d, 12b). The posterolateral and posteromedial amygdalar cortical nuclei show a slightly more abundant population of *Sst* cells (PLCo, PMCo; Fig. 12b).

As regards the subpallium, the striatum population of *Sst* cells develops over these stages (E15.5, E16.5) a nearly mature appearance. The distribution remains gradiental along the radial dimension extending from the olfactory tuberculum, past the ventral striatum, into the main body and its subventricular zone at E15.5, (St, OT, VSt; Fig. 11a–f), but seems denser and more uniform at E16.5 (St; Fig. 12c–h). In contrast, there are few *Sst* cells in the fundus striati (under the tag ‘l’ in Fig. 12g, h). Interestingly, horizontal sections illustrate a relatively high density of *Sst* cells along the IPAC primordium (interstitial nucleus of the posterior limb of the anterior commissure), at the transition between dorsal and ventral striatum regions (IPAC; Fig. 12d–f). This aggregate encloses the label-free posterior limb of the anterior commissure and limits caudomedially with the largely unlabeled pallidal intermediate stratum forming the GP (GP; Fig. 12e–h).

Found along the radial dimension of the Pal domain extending past the GP into the subpial olfactory tubercle, the ventral pallidum (VPal) shows increased presence of *Sst* cells compared to GP, though always less than the neighbouring striatal and diagonal superficial domains (GP, VPal, VSt, SI; Figs. 11e, f, 12c, d). The periventricular pallidal stratum (identified by NKX2.1-immunoreaction in Fig. 11) is represented by the BSTL primordium, found lateral to the *Sst*-positive BSTM (which is largely negative for NKX2.1). The supracapsular BSTL is practically devoid of *Sst* cells, particularly at its subventricular zone, though some *Sst* cells characterize the local mantle deep to the internal capsule (BSTLsc; Fig. 11b–d). The related pallidal paraseptal area, identified here as SePal, similarly shows a number of dispersed *Sst* cells within the deeper mantle zone, next to its negative subventricular zone (SePal; Fig. 11e, f). These deep elements may represent remnants of the earlier deep migration stream across the Pal into the striatum, and are in fact clearly continuous with the striatal population (Fig. 11c, d). Finally, it is possible that the amygdaloid end of the pallidal mantle is represented by the medial part of the CA complex, which appears scarcely populated by *Sst* cells, in contrast with the richly populated lateral part of CA (CeL, CeM; Fig. 11b). This difference was also observed in the adult CA (Fig. S3b). Collateral data mining in the Allen Developing Mouse Brain Atlas revealed that *Isl1* expression in the CA seems restricted to its medial part (CeM).

The diagonal domain appears segregated radially into deep, intermediate, and superficial components, like the pallidal domain. All of them contain abundant *Sst* cells. The periventricular derivative is the BSTM, which appears largely as a thin strongly *Sst*-positive band in sagittal sections (Fig. 11), but seems broader in horizontal sections (Fig. 12). In fact, horizontal sections suggest that this population of *Sst* cells is dual, being composed by an anterolateral thin band of large and strongly *Sst*-expressing neurons (marked by an asterisk in Fig. 12h) and a caudomedial broader parallel band of smaller and less strongly labeled cells; outside the asterisk-marked cells in Fig. 12h). This dual constitution was observed as well at the amygdaloid and paraseptal ends of the BSTM formation (unlabeled in Fig. 11; BSTMa, BSTMps; Fig. 12), and probably accounts for some of the detailed subdivisions described there in the adult (see “Discussion”). The BSTM complex can be subdivided along the septo-amygdaloid axis into paraseptal, central (supracapsular), and amygdaloid parts (BSTMps, BSTMsc, BSTMa; Figs. 1b, 11b–f, 12c–h). The paraseptal BSTM progressively diminishes in cell number toward the negative preoptic area (e.g., Figs. 11f, 12c). Adjacent laterally to the BSTMa, there is the very dense and highly labeled primordium of the lateral part of the CA complex (CeL; Fig. 11b), which may be understood as representing the last periventricular diagonal formation along the septoamygdaloid axis. The CeL is continuous superficialward with the DgMC nucleus (usually named ‘magnocellular preoptic nucleus’ in the literature, though it obviously lies outside the preoptic region), which represents the amygdaloid intermediate Dg stratum, found lateral to the substantia innominata (DgMC; Fig. 11b). At the intermediate stratum of the central Dg area, there appears immediately under the internal capsule a small caudomedial extension of the unlabeled GP, whose cells are distinctly NKX2.1-immunoreactive; we identified the two GP parts identifiable in this material as corresponding to the prospective external and internal pallidal segments (EGP, IGP; Fig. 11b–d; note the estimated pallidal boundaries are highlighted by black lines); it is unclear whether the IGP originates primarily at the Pal or Dg domains; in any case, it shows few *Sst* cells, which is a Pal-like feature. Underneath the IGP there appears the substantia innominata (SI), another constituent of the central diagonal intermediate stratum, which displays a dispersed population of *Sst* cells (SI; Fig. 11b–f). At the superficial stratum of the diagonal domain there appears the diagonal band, whose horizontal nucleus shows a contingent of *Sst* cells (HDB, DB; Figs. 11b–f, 12a–c). The medial septal surface also shows a *Sst*-positive population, which may correspond to the prospective vertical diagonal band nucleus (Se; Fig. 12c–e).

## Discussion

### Diversity of subpallial sources of tangential migrations and the associated terminology problem

In classical models, the subpallium was composed exclusively of striatal and pallidal parts. In recent times, the subpallium model was expanded to include the preoptic area (previously ascribed to the hypothalamus; see Shimogori et al. 2010; Puelles et al. 2010) and the hemispheric stalk (peduncular) region or diagonal area (Fig. 1a, b); the latter was known previously either as substantia innominata, or as anterior entopeduncular area; Bulfone et al. 1993, 1995; Puelles et al. 2000, 2004, 2013). Among the molecular characteristics that unify these subpallial regions is the overall early expression of *Dlx* family, *Mash1* and *Arx* genes (Puelles et al. 2004; Shimogori et al. 2010). Analysis of gene markers that label differentially a particular subpallial ventricular zone, such as *Nkx2.1* in the pallidum, stalk region and preoptic area, and *Shh* in the POA1 part of the preoptic area (Fig. 1b), indicated that all major subpallial divisions extend from the septum to the amygdala, along the oblique septo-amygdaloid axis (Swanson and Petrovich, 1998; Puelles et al. 2000, 2013; Flames et al. 2007; Medina and Abellan 2012). The apparent caudal pole of this subpallial complex represents the caudal ganglionic eminence, ascribed to the amygdala, to which may be added the preopto-hypothalamic transition area (CGE, POH; Fig. 1a, b; Bulfone et al. 1993; Puelles and Rubenstein 1993; Xu et al. 2004, 2008; Butt et al. 2005; Fogarty et al. 2007; Sousa et al. 2009; Lee et al. 2010). Note the lateral ganglionic eminence (LGE) largely corresponds to the striatal domain, whereas the medial ganglionic eminence (MGE) encompasses the pallidal domain, the diagonal area, and part of the preoptic area (Fig. 1b).

Flames et al. (2007) mapped at least 18 molecularly distinct progenitor domains at the subpallial ventricular/subventricular zone (Fig. 1c; note these are mostly aligned parallel to the septoamygdaloid axis). It was concluded that these domains might represent as many independent sources of specific neuronal types, which originate partly a diversity of tangentially migrating populations, and partly discrete radially stratified populations of the local mantle zone (i.e., a theoretical minimum of 18 × 3 = 54 cell populations, counting periventricular, intermediate and superficial strata at each subpallial domain; e.g., lateral bed nucleus striae terminalis, globus pallidus, and ventral pallidum plus pallidal olfactory tuberculum, within the pallidum). This conceptual background significantly qualifies the earlier simpler concepts of the lateral, medial, and caudal ganglionic eminences (LGE, MGE, and CGE), which were initially thought to represent homogeneous histogenetic entities. Indeed, classic embryologic studies had simplistically assimilated the striatum to the LGE, and the pallidum to the MGE.

The subpallial region that occupies the telencephalic stalk area, intercalated between the preoptic area and the pallidum, was recently renamed diagonal area (Dg) in the Allen Developing Mouse Brain Atlas (online since 2009) and Puelles et al. (2013). This region is stretched like its neighbours along the septo-amygdaloid axis (Fig. 1b, c). Its intermediate stratum formed by dispersed neurons was classically identified as the ‘substantia innominata’ (which contains the cholinergic neurons of Meynert’s basal magnocellular nucleus, among other elements, and merges laterocaudally with the ‘sublenticular extended amygdala’). The Dg subpial stratum is occupied by the ‘diagonal band nuclei’ (horizontal and vertical), which lie interstitial to the diagonal (amygdaloseptal) tract and end at the medial septum; these nuclei also display in late embryos and the adult a prominent cholinergic population (Zaborszky et al. 2012; Allen Developing Mouse Brain Atlas). Various reports concluded that these cholinergic neurons, dependent on *Lhx7/8* and *Isl1* are not produced locally within the Dg domain (understood as medial extended amygdala, or MGEcv area; Zhao et al. 2003; Elshatory and Gan 2008; Medina and Abellan 2012); moreover, they do not share origins with other telencephalic cholinergic neurons presumedly produced at the preoptic area. Interestingly, our data reveal that ventricular and mantle expression of *Lhx7/8* is strictly restricted to the Pal and Dg areas, whereas the preoptic region only shows a few positive cells in its mantle, possibly migrated in from the Dg (Fig. S1). We believe this interpretation is consistent with corresponding data shown by García-López et al. (2008; their Figures 1C, 4D; note also the neurons expressing ChAT illustrated therein only overlap the Dg region). In our opinion this weakens the hypothesis of a preopto-commissural origin of cholinergic neurons (since the differentiation of these cells needs *Lhx7/8* signal; Zhao et al. 2003). Here we merely wish to emphasize that whatever their origin may be (see also below our discussion of Pombero et al. 2011), these cells selectively mature within the diagonal band nuclei, as well as within the substantia innominata, both of which are components of the Dg. The diagonal histogenetic area also encompasses a periventricular stratum that includes the medial part of the stria terminalis complex (BSTM; note the adjoining BSTL part—which differentially expresses *Isl1* [Allen Developing Mouse Brain Atlas]—is instead pallidal; Fig. 1d). The Dg was characterized by Flames et al. (2007) as a molecularly distinct areal component of the MGE, identified as the pMGE5 subdomain (see Fig. 1b, c). This domain was the only ‘pallidal’ (meaning MGE) area that expressed the transcription factor *Er81* (Flames et al. 2007; their Figure 2D); it also shows ventricular patches of *Shh* signal which contrast with the massive POA expression (Flandin et al. 2010; present results). As mentioned above, the pallidal BSTL region expresses differentially *Isl1*, compared to the diagonal BSTM.

The Dg was previously named ‘anterior entopeduncular area’ in early reports on the prosomeric model (AEP; e.g., Bulfone et al. 1993; Puelles and Rubenstein 1993; Rubenstein et al. 1994). The AEP name has since been used widely, though it proved to be imprecise (and thus inconvenient), since it suggests an isolated cell population interstitial to the medial or lateral forebrain bundles, rather than a complete radial (ventriculo-pial) histogenetic domain, as was intended. Puelles and collaborators thus came to regard their own term as obsolete. In the search for a better alternative name, the momentary absence of markers distinguishing this area from the pallidum led to the idea that this area could be seen as a part of the Pal, or, at least, of the MGE. Alternative names accordingly employed in recent literature include ‘caudal and medial MGE’ (Nery et al. 2002; Legaz et al. 2005), ‘central and ventral MGE’ (Fogarty et al. 2007), ‘anterior peduncular area’ (García-López et al. 2008), ‘ventral MGE’ (Flandin et al. 2010, 2011), and ‘caudoventral MGE’ (Bupesh et al. 2011a, b; Medina and Abellan 2012). This excess of options generates by itself considerable semantic confusion, since none of these authors provided a subpallium map showing the precise location and extent of this area. We think that all these names are similarly inconvenient. First, because the axis of reference for the diverse positional descriptors used remains undefined and vague (probably the ‘central’ and ‘caudal’ terms allude to the arbitrary anteroposterior sequence of coronal section levels –the axis of the microtome- which is devoid of true morphologic value in the prosomeric model, but they might refer instead to the oblique, more realistic septoamygdaloid axis), and, secondly, because the precise position of the ‘medial’ or’ventral’ area along the dorsoventral dimension also seems unclear (no defining landmarks). Confusingly, the locus of this area is often implied to be circumscribed to a limited areal spot (most authors identify it only at a standard coronal section level), though the model shown in Fig. 1b, c suggests that the relevant distinct stalk area actually extends all the way from the septum into the amygdala (Puelles et al. 2013; see text of Figure 1d). Accordingly, the position within the MGE complex of this specific area and the developmental phenomena associated to it probably result imperfectly understood by most readers.

Curiously, Flames et al. (2007) resolved minimally the molecular distinction of the stalk (Dg) histogenetic area relative to the pallidum, observing that the former selectively expresses *ER81 (Etv1)* and lacks *Couptf1* signal, whereas the opposite is true for the pallidum. Present data illustrate a remarkably precise correlation of *Sst* neurons with the mantle of this domain; García-López et al. (2008) previously underlined the restricted presence at the same locus of calbindin-positive neurons, streaming tangentially into the pallial amygdala in a similar way as we observed *Sst* cells. Therefore, it is both possible and convenient (and is supported by the present results) to have a distinctive name for the stalk area, leaving aside the old term AEP, as well as the vague descriptors of its position within the MGE. The recently updated prosomeric model (L.P., reference atlases developed for the Allen Developing Mouse Brain Atlas; http://www.developingmouse.brain-map.org; Puelles et al. 2012, 2013) aimed to resolve this problem by proposing for the stalk area the alternative term *diagonal area* (Dg); this conveys the correct topographic linear extent along the oblique hemispheric stalk and septoamygdaloid axis, and is based on a well-known classic concept and related identifiable surface landmark, the diagonal band (i.e., the subpial diagonal band formation generally forms a surface relief that separates the flat preoptic surface from the olfactory tuberculum, which represents the neighboring subpial portion of the Pal and St). This name refers to the adult region and corresponding characteristic cell populations, irrespective whether these are produced or not within the Dg proper (e.g., case of the cholinergic cells commented above).

In summary, the narrow diagonal radial histogenetic domain defined at the hemispheric stalk (Dg; Fig. 1d) is traversed orthogonally by the medial and lateral forebrain bundles, and encompasses the diagonal band nuclei superficially (jointly with the diagonal band tract), the innominate/magnocellular basal populations at the intermediate stratum (with the ventral amygdalofugal tract), and the medial bed nuclei of the stria terminalis complex (BSTM) periventricularly (with part of the stria terminalis tract). At its septal end, the Dg is continuous with the medial septum via the vertical limb nucleus of the diagonal band. At its opposite amygdalar end, our data suggest that the Dg finishes at the amygdalar BST nucleus (BSTA) and the associated lateral part of the central nucleus (CeL; see below).

### Antecedents of the *Sst* cell migration

In this report, we study the apparent radial origin and subsequent wide tangential migratory distribution of *Sst* cells in the mouse telencephalon between E10.5 and E16.5. In the final distribution such cells populate within the pallium the whole cerebral cortex (a well-studied point we do not need to examine in detail), the claustrum and the pallial amygdala, showing scarce contributions to the olfactory bulb. *Sst* cells also invade the subpallium, though differentially. They are massively present in the lateral part of the central amygdaloid nucleus, as well as in the medial part of the bed nucleus stria terminalis complex, both of which are understood here as intrinsic (non tangentially migrated) derivatives of the diagonal area (see Suppl. Fig. 4; Puelles et al. 2013, and reference atlases of the Allen Developing Mouse Brain Atlas, online since 2009); there is also a labeled subpopulation of striatal interneurons, many labeled cells throughout the olfactory tuberculum, and some weakly labeled cells within the pallidal BSTL. In contrast, *Sst* cells eschew rather selectively the globus pallidus and the preoptic area. Our results suggest that the earliest, and possibly the main, origin of telencephalic *Sst* neurons is the diagonal area, which is a longitudinal hemispheric stalk subregion of the medial ganglionic eminence that appears intercalated between the pallidum and the preoptic area. The diagonal area (Dg) roughly corresponds to the pMGE5 ventricular progenitor domain of Flames et al. (2007).

Somatostatin neurons were previously mapped immunocytochemically in rat and mouse embryos (e.g., Shiosaka 1992; García-López et al. 2008; Real et al. 2009). Compared to these earlier studies, our broader in situ hybridization analysis in different section planes and more embryonic stages provides much additional detail about their development, particularly because we explored in adjacent sections the relationships of *Sst* cells with the subpallial expression domains of several transcription factors (*Gbx2*, *Dlx5*, *Lhx7*-*8*, *Nkx2.1*, *Nkx5.1*), as well as with transcripts of *Shh*, the gene coding the morphogen SHH, significantly expressed in a large population of preoptic cells that invade the pallidum (Flandin et al. 2010). In agreement with Sousa et al. (2009), we detected the first telencephalic *Sst* cells at E10.5. In contrast, Shiosaka (1992) first illustrated such cells in E16.5 rat embryos (equivalent to E14.4 mouse embryos; Clancy et al. 2001). Similarly, Garcia-López et al. (2008) only reported data in E14.5 mice, while Real et al. (2009) saw scattered cells in the mouse Dg at E11.5. These are all immunocytochemical studies.

Authors who examined the origin of cortical *Sst* cells via transgenic and in vitro approaches generally concluded that these originate from a *dorsal* pallidal part of the MGE (see specific citations below), whereas García-López et al. (2008), Real et al. (2009) and Medina and Abellán (2012) associated the origin of *Sst* cells that invade the amygdala to the anterior entopeduncular area or *MGEcv* (i.e., the Dg), in agreement with our present conclusions. In the following sections, we will comment on the development of telencephalic *Sst* cells, and discuss the conflicting views about the origin of these cells.

### Early development of *Sst* cells

In the present report, we mapped descriptively the progressive changes observable in the topography of *Sst* cells in the developing mouse telencephalon. Given the existence of several experimental transgenic tracings demonstrating that such cells migrate tangentially from a restricted (single) subpallial source into other telencephalic domains (see citations below), we have parsimoniously interpreted the observed absolute changes in topography as evidence of tangential migration, starting at the apparent source (i.e., where the earliest cells are found). Our results revealed that the earliest *Sst* cells identified at E10.5 are not uniformly distributed within the MGE, being restricted to a thin radial domain of the MGE mantle zone that appears intercalated between the pallidal and preoptic regions, as identified by differential markers. This is precisely the estimated position of the Dg (Puelles et al. 2013; Fig. 1b) and roughly correlates with the ventricular pMGE5 domain of Flames et al. (2007). Our correlative mappings of diverse subpallial markers at E10.5 and E11.5 showed that the early *Sst* population does not overlap either with the massive subpopulation of *Gbx2* neurons that appears restricted to the pallidal area, nor with *Nkx5.1*-expressing preoptic neurons. We detected also no significant overlap between early *Sst* cells and the sizeable stream of *Shh*-positive cells that migrates tangentially from the preoptic area into the pallidal mantle. Nevertheless, we systematically observed that the Dg ventricular zone itself shows patchy *Shh* expression, a pattern that is distinct from the massive expression observed at the nearby preoptic POA1 ventricular zone; a small parallel contribution of Dg to the *Shh*-positive population in the subpallial mantle seems thus possible, though such cells would not overlap the Dg-derived *Sst* population (possible salt-and-pepper pattern, separately also implied in the hypothesis that some cholinergic poulations are locally produced). The subpallial nature of the diagonal *Sst* cell population is corroborated by the shared expression of general subpallial markers such as *Dlx5*, *Nkx2.1,* and *Lhx7/8*.

Already at E10.5, it can be seen that the Dg radial stream of *Sst* cells is continuous superficially with an incipient subpial aggregate of similar cells spreading tangentially lateralwards; at this stage, these subpial cells partially cover marginally the pallidal mantle core occupied selectively by *Gbx2*- and *Shh*-positive cells. A few of these subpial *Sst* elements even penetrate the striatal marginal stratum (identified as the subpallial mantle zone devoid of the *Nkx2.1* pallidal marker). This *superficial subpallial migration stream* (abbreviated as SSpM) becomes much better developed at subsequent stages (E11.5-E14.5). It clearly represents the main pathway for the arrival of diagonal *Sst* cells at the striatum (complemented by outside-in radial invasion from the SSpM) and the pallium (first traversing subpially the prospective layer III stratum of the olfactory cortex primordium, which transiently lies at the brain surface at these early stages; Valverde and Santacana 1994); *Sst* cells then enter the marginal stratum of the insula and proceed into the isocortex in a gradiental pattern (the hippocampus and entrorhinal cortex seems to be invaded through an analogous caudal marginal stream across the amygdala). Our data indicate that the alternative intermediate and subventricular migration pathways so common for other subpallial migrating cortical interneurons (Anderson et al. 2001; Marín and Rubenstein 2003) are of minor importance in the case of the *Sst* cells. In contrast, a subventricular tangential migration route seems relevant for the invasion of the pallial amygdala, in addition to the SSpM (compare Wang et al. 2010; their Fig. 2).

Though the SSpM first crosses the pallidal marginal stratum at the locus of the prospective pallidal olfactory tuberculum (where a subpopulation persists at later stages), the pallidal mantle core remains practically devoid of *Sst* cells at all stages, as is true as well for the mature globus pallidus and, partly, for the lateral BST formation (also pallidal in nature). This apparently reveals a persistent non-permissive or repellent pallidal effect on the migrating *Sst* cells; this phenomenon may explain as well the paucity of *Sst* cells that migrate subventricularly into the striatum and the cortex, since they need to cross the pallidal territory. Moreover, no *Sst* cells were ever observed within the preoptic area; this result indicates a clearcut spatial orientation of the SSpM in the opposite palliopetal direction, possibly influenced by repellent signals spreading out of the preoptic area. It may be speculated whether SHH highly present in the preoptic and pallidal environments is repulsive for the *Sst* cell population (compare Xu et al. 2010).

### Ulterior migration and development of definitive *Sst* cell populations

Independently of the *Sst* cells that migrate tangentially away from the Dg via the SSpM and the deep and superficial amygdalar streams, the Dg histogenetic area also develops radial derivatives that mature locally; these appear thereafter clearly intercalated in the subpallial mantle between the *Sst*-negative pallidal and preoptic areas. This locus corresponds to a radial domain placed obliquely along the telencephalic stalk, which has received little embryological attention *per se* so far, but is known to exist since the nineties (the anterior entopeduncular area or AEP of Bulfone et al. 1993). This locus also shows characteristic structure in the adult. Previous morphologic analysis suggested that the Dg superficial stratum contains the bed nuclei of the diagonal band, extending obliquely (diagonally) from the amygdala to the medial septum. In its turn, the Dg intermediate stratum deep to the diagonal band forms the classic substantia innominata (which encompasses a major part of the cholinergic basal magnocellular nucleus of Meynert, but also contains other cell types). Finally, the corresponding Dg periventricular stratum contains what can be defined as the medial BST formation. The literature contains a clear idea of medial and lateral BST regions (e.g., in De Olmos et al. 1985), but confusingly also includes a diversity of classifications of individual BST nuclei into these main compartments, but we do not need to discuss these in detail here (compare, for instance, Ju and Swanson, 1989; Ju et al. 1989; Walter et al. 1991; De Olmos et al. 2004; Medina and Abellan 2012). Puelles et al. (2013) suggested, and we now corroborate, that the lateral BST formation (BSTL) is pallidal, whereas the medial BST (BSTM) is diagonal, as noted in terms of relative abundance of *Sst* neurons (see Walter et al. 1991 for the human brain) and differential expression of *Isl1* (only in BSTL). The BSTM also includes some supra- and subcapsular elements classified by some authors within the medial extended amygdala (e.g., Medina and Abellan 2012). Note Medina and collaborators already interpreted part of the extended amygdala as a radial derivative of the Dg, identifying it as ‘entopeduncular area’ in Garcia-Lopez et al. (2008), or as ‘caudoventral MGE’ in Medina and Abellán (2012). The ‘extended amygdala’ concept, which is supported by the present results, essentially underlines the developmental sharedness of molecular, histogenetic, and other (e.g., hodologic) properties along the septo-amygdaloid complex of subpallial areas (Fig. 1a–c), as was realized by Heimer himself (personal communication to LP).

We cannot postulate from our material that all Dg derivatives express *Sst*, though we presently do not know of other selective markers of potential Dg mantle derivatives other than *Chat*, which identifies cholinergic neurons. At the most advanced developmental stage examined by us (E16.5) we saw rather sparse *Sst* cells at the level of the diagonal band nuclei, together with a rather dispersed *Sst* population within the innominate area deep to them; in contrast, there appears a well-developed population of large and small *Sst* cells centered within the periventricular BSTM formation. Importantly, the latter population was found to lie just outside the pallidal domain expressing *Nkx2.1* (or NKX2.1) from E13.5 onward (apparently due to down-regulation of initial *Nkx2.1* expression at the Dg), while it clearly lies within the *Dlx5*-expressing subpallial mantle (Figs. 9i, l, 11 and S2); this observation was unexpected, since it is widely accepted in the field that the whole MGE (including Pal, Dg and POA) initially expresses *Nkx2.1*, and it was assumed that this state was permanent throughout the MGE. There exists as well a parallel supracapsular migratory stream of hypothalamic *Otp*-positive neurons that approach the medial amygdala (Wang and Lufkin 2000; Garcia-Moreno et al. 2010; Morales-Delgado et al. 2011). We checked whether this periventricular *Otp* population coincides with the *Sst* one at the telencephalic stalk However, the *Otp* stream uses the thin *pallialcorridor* that directly connects the alar hypothalamus with the pallial amygdala (topologically caudal to the subpallium as a whole), and therefore does not coincide with the *Sst*-positive BSTM; it demonstrably passes just outside the *Dlx5*-positive domain that contains the subpallial *Sst* BSTM elements (Figs. 9i, l, 11b–f; S2a–c).

The cholinergic neurons of the diagonal band and the substantia innominata (basal magnocellular nucleus) probably represent a significant part of the *Sst*-negative intermediate and superficial Dg mantle (as suggested by material shown at the Allen Adult Mouse and Developing Mouse Brain Atlases, or García-López et al. 2008). Given the recent unexpected notion that many of these cells (but not all) originate distantly in the ventral pallium and belong to a *Tbr1*-expressing lineage that invades the Dg (Pombero et al. 2011), it is open to discussion how far the few *Sst* cells present in these superficial Dg areas may represent the local intrinsic diagonal population, in contrast with local and immigrated cholinergic cells. On the other hand, Zhao et al. (2003) reported a dependency of the subpallial cholinergic cell type on *Lhx8* gene expression, a pattern that is restricted to the Pal and Dg regions of the MGE (as was corroborated by present data; Fig. S1). These authors just assumed a local subpallial origin of the cholinergic cell type, without commenting on the potential role of diverse MGE progenitor subareas in their production, an issue subsequently underlined by Flames et al. (2007). The demonstrated requirement of *Lhx8* signal in the MGE for the local differentiation of cholinergic neurons (though these potentially may originate elsewhere) may bespeak of an indirect non-cell-autonomous effect due to local MGE mantle conditions controlled by *Lhx8*. According to the results of Pombero et al. (2011) such an effect may act upon cells previously produced in the pallium under control of *Tbr1* and secondarily migrated tangentially into the diagonal part of MGE. If this were not so, we would expect cholinergic cells differentiating massively everywhere the *Lhx8* signal appears in the subpallium, that is, in the whole Pal and Dg domains, which is not the case. This hypothesis accordingly may conciliate the data of Zhao et al. (2003) and Pombero et al. (2011). The fact that most basal cholinergic neurons finally populate the Dg area (diagonal band nuclei and basal magnocellular population within the substantia innominata) suggests a differential local microenvironment (more specific than is allowed by the full domain of *Lhx8* expression), and further supports the notion that the Dg is a molecularly distinct from the Pal (Flames et al. 2007), as is also indicated by the restricted local downregulation of *Nkx2.1* and the selective production of *Sst* cells. Interestingly, Zhao et al. (2003) observed that *Sst* cells were not affected by the null mutation of *Lhx8*.

The supracapsular BSTM arch that forms at amygdaloid, central, and paraseptal periventricular regions of the Dg area (terminology of Puelles et al. 2013; Fig. 1b) appears well populated by *Sst* cells from E14.5 onward; it is first visible as an emergent aggregate of *Sst* cells at E13.5 (note Bayer, 1987 found in the rat birthdates between E15 and E17 for what Puelles et al. 2013 call the ‘paraseptal BSTM portion’; these rat stages correspond to E13.6–14.8 in the mouse; Clancy et al. 2001). The rather compact *Sst*-positive BSTM population stands out from the largely *Sst*-negative pallidal BSTL formation, which overlies the globus pallidus (incidentally, we think that our observation that external and internal globus pallidus parts can be distinguished according to their topography relative to the *Sst*-positive BSTM arch—the latter covers directly the IGP (see Fig. 11)—may be aclaratory with respect to the traditional concept that the IGP originates in the hypothalamus). The BSTL locus only transiently shows a few *Sst* cells that migrate tangentially through the pallidal subventricular stratum into the striatum (or the pallium). The BSTM supracapsular arch extends across the paraseptal Dg area (which neighbours the crossing fibers of the anterior commissure) into the diagonal part of the subpallial medial septum, where individual parts of the BSTM formation have been recognized in adult rodents (these are often described as ‘anterior BST’ in the relevant literature). The amygdaloid BST nucleus is wholly or in part a caudal prolongation of the supracapsular BSTM into the amygdaloid part of the subpallium (Puelles et al. 2013; Fig. S4).

The potential existence of intrinsic amygdaloid Dg derivatives—and *Sst* cells—that are not tangentially migrated from outside the amygdala proper was apparently never considered previously. The existence of an amygdaloid diagonal subpallial subdomain first appeared defined in the Allen Developing Mouse Brain Atlas (online since 2009; see also Puelles et al. 2013). Our present results strongly suggest that at least a significant aggregate of *Sst* neurons later found within the *lateral* subregion of the central amygdaloid nucleus, which is widely accepted as a part of the subpallial amygdala, derives primarily via radial migration from the amygdaloid pole of the Dg progenitor area (CeL in Fig. S3A, B; Ce in Fig. S4; the medial and capsular parts of the central amygdalar nucleus may be pallidal or striatal in origin). Bupesh et al. (2011a) reported a tangentially migrated contribution to Ce of more rostral parts of the Dg domain (their MGEcv area), but these experiments did not include labeling of the amygdalar end of the Dg complex, and were generally performed at E14.5, which we believe is too late to detect the radial migration we deduce forms the CeL; we start to observe this primordium already at E12.5. The presence of appropriately oriented radial glia fibres across the Ce is shown in our Fig. S4. The diagonal magnocellular nucleus (DgMC; known in the literature as ‘preoptic magnocellular nucleus’) seems also an intermediate stratum derivative of the amygdaloid Dg subdomain. In our identification of this cell group in a position lateral to the locus of the horizontal diagonal band nucleus we followed standard rodent atlas advice (Watson and Paxinos 2010; note Paxinos and Franklin 2013 identified this nucleus as ‘lateral nucleus of the diagonal band’, considering as we do that it is not preoptic in nature). As is shown in several of our Figures (Figs. 1b, 8, 10p, q, 12), our data indicate that the diagonal amygdala contacts directly the striatal amygdala behind the caudal end of the pallidal amygdala (the striatal amygdala is mainly a caudal part of the dorsal striatum, e.g., according to *ER81* and *Six3* expression). This arrangement had not been disclosed so far.

The lateral central amygdaloid subnucleus (CeL) is a compactly *Sst*-positive population identifiable as an incipient radial migratory stream at the amygdaloid Dg subarea already at E12.5 (asterisk in Fig. 7f, g). This mass later appears systematically intercalated between the striato-pallidal complex and rostral parts of the pallial amygdala (L/BL primordia); it characteristically preconfigures the future CeL nucleus from E13.5 onward (Figs. 8e–g, 9a, 10g–j, p, 11a, 12c–f). We did not find any reference to this particular subpallial primordium in the embryologic literature, nor had we been aware ourselves of its existence previously. Representative sections shown in our Figs. 10 and 12 illustrate the histogenetic and genoarchitectonic continuity of the CeL primordium with the supracapsular BSTM arch, providing a novel insight into the development of this area. In contrast, Medina and Abellán (2012) recently interpreted that *Sst* cells reach the central amygdala via tangential migration from the ‘medioventral MGE’ (equivalent in their schemata to our paraseptal and/or central Dg; see comments above about similar conclusions of Bupesh et al. 2011a). The amygdaloid end of the BSTM component of Dg also apparently builds separately the well-known amygdaloid BST nucleus (BSTA). We thus believe this would represent likewise a radially migrated Dg-derived entity, rather than a result of tangential migration.

On the other hand, numerous tangentially migrating *Sst* cells invade the *pallial* amygdala, either via the subpial SSpM, or via a specific, well-developed, amygdaloid subventricular migratory pathway. We could not assess whether these cells originate specifically from paraseptal, central, or amygdaloid parts of the Dg area, or come from all of them. A feature suggesting a general Dg origin is that the earliest cells invading the pallial amygdala via the SSpM were observed at E11.5 (Fig. 5ae, af), and this pathway was still very distinct at E12.5 (superficial arrow; Fig. 7e, f). The subventricular subpallial migratory stream (SvSpM) that likewise targets the pallial amygdala appeared at E12.5 in a relatively more dorsal (perhaps more origin-selective) position (SvSpM; deep arrow; Fig. 7g–k). It is possible that some of these deep SvSpM cells reach the overlying cerebral cortex (entorhinal and hippocampal areas, and perhaps even occipitotemporal areas) via a transamygdalar route. Both streams are less distinct at E13.5 and subsequent stages; this later period is characterized by a substantial invasion by *Sst* cells of the lateral (L), basomedial (BM) and amygdalohippocampal (AHi) amygdaloid nuclear primordia. There is the apparent exception of the basolateral, posteromedial cortical, and medial amygdaloid nuclei, which stand out as loci with few *Sst* neurons (BL, PMCo, MePV, MA; Figs. 10, 11, 12).

At E16.5, the L primordium appears densely populated and even delineated by *Sst* cells (Fig. 12f–h). Horizontal sections show that the rostral tip of L roughly coincides with the locus where the fibers of the anterior commissure reach the amygdala. Similar numbers of *Sst* cells label the IPAC nucleus, a part of the extended amygdala (interstitial nucleus of the posterior limb of the anterior commissure; Fig. 12d–f). More rostrally, along the line where the anterior limb of the anterior commissure enters the external capsule (lateral to the striatum) a distinct laminar population of *Sst* cells is visible which appears in a similar place as the L nucleus, but lies rostral to it, and is much thinner. We think that it corresponds to the primordium of the bed nucleus of the external capsule (BEC), a formation derived from the ventral pallium that was recently distinguished at this locus (Puelles 2014); this primordium probably also corresponds to the supposedly transient ‘reservoir’ of Bayer and Altman (1991). The amygdalar L nucleus is held to be a ventropallial derivative on genoarchitectonic grounds, whereas the BL was ascribed to the lateral pallium (Medina et al. 2004). There accordingly appears to exist a preferential invasion of ventropallial cell masses by the tangentially migrated *Sst* cells as they pass beyond the pallio-subpallial boundary (this includes the large population invading layer III of the piriform cortex, which also is an integral part of the ventral pallium (Puelles 2014). At postnatal stages, further development of the pallial amygdaloid nuclei and their neuropil leads to substantial dilution or decrease by cell death of the contained population of *Sst* cells, though L still retains in the adult more *Sst* cells than the BL nucleus (compare L and BL in Fig. S3b).

Our analysis of the invasion of the isocortical plate by *Sst* cells was largely centered on the chronology of the arrival of these cells to the different areas. By E16.5 the whole cortex was covered superficially by *Sst* cells. As mentioned above, we believe that the main pathway for this tangential migration is the SSpM. Following Puelles (2014), we interprete that marginal *Sst* cells observed over the cortical plate up to E14.5 result subsequently distributed to the subgranular cortical layers (since the cortical plate at E14.5 is largely formed by the prospective layer 5 and layer 6 pyramids). As was previously reported, the major adult population of cortical *Sst* interneurons has a subgranular topography (see Fig. S3b). Presumably, labeled embryonic cells occupying a similar marginal position at E15.5 and E16.5 will be distributed to the sparser population later found in the supragranular layers.

### Telencephalic subpallial domains and the origin of *Sst* cells

A subpallial origin of most inhibitory cortical interneurons in the mouse is supported by the fact that practically all cortical interneurons in mice derive from the *Dlx5/6*-expressing subpallial lineage (Stuhmer et al. 2002), and such cells are not part of the massive complementary pallial *Emx1*-expressing lineage (Iwasato et al. 2000; Gorski et al. 2002). Whereas some pallial explants do give rise to some cells producing GABA in vitro (Götz et al. 1995; He et al. 2001; Bellion et al. 2003; Nery et al. 2003), it is so far unclear whether these results are extrapolable to the in vivo situation (they may result alternatively from differences in genomic regulation created by in vitro conditions, or from a potential initial content of migrating cells of subpallial origin capable of spontaneous or stimulated proliferation in the explanted tissue). In humans, Letinic et al. (2002) reported that more than half the population of cortical inhibitory interneurons derives from mitoses occurring within the pallial subventricular zone (but see Ma et al. 2013).

Our results corroborate previous data suggesting that SST-positive cells reaching the amygdala derive from the Dg domain (old AEP; García-López et al. 2008; Real et al. 2009; Bupesh et al. 2011a, b). We provide here a more detailed description of the relationship of the Dg area with *Sst* neuron production and migration, which leads us to suggest a Dg origin for most cells expressing *Sst* in the telencephalon. This seems partly contradictory with some earlier experimental results, which suggested that the major origin of such cells is the pMGE1 sector of the pallidum, which clearly does not form part of the Dg (Fig. 1c). We think the analysis of this issue (and other analogous issues pertinent to the subpallium) improves by translating the diverse contributions into the comprehensive conceptual framework developed for the subpallium in the Allen Developing Mouse Brain Atlas (reference atlases, online since 2009; www.developingmouse.brain-map.org); see also Puelles et al. (2013). This model allows striatal, pallidal, diagonal, and preoptic alternative origins of the *Sst* cells to be visualized (Fig. 1a, b), and diverse septal, paraseptal, central and amygdaloid sectors along these domains can be located rather precisely with reference to the septo-amygdaloid axis, as well as periventricular, intermediate and superficial strata with regard to the radial dimension.

#### Striatal origins

A striatal origin of *Sst* cells can be dismissed straightforwardly, because the cerebral cortex of *Nkx2.1*^−*/*−^ mutants, which essentially lose all MGE progenitor fates, and develop a larger striatum (Pal, Dg, and POA are repatterned as striatum-like domains; Sussel et al. 1999), contains practically no interneurons expressing *Sst* at E18.5 (Anderson et al. 2001). Additionally, primary cultures testing postnatal development of interneuron subtypes in the *Nkx2.1*^−*/*−^ mutant cortex showed absence of somatostatin and parvalbumin interneurons (Xu et al. 2004).

#### Pallidal versus diagonal origins

Transgenic mice lines expressing GFP or LacZ reporters in the *Nkx2.1Cre*-labelled lineage were expected to show co-labeling of all SST-positive cortical interneurons. Surprisingly, many parvalbumin- and SST-positive interneurons did not show the reporter, particularly in superficial cortical layers of *Nkx2.1Cre:Z/EG* and *Nkx2.1Cre:R26R*-*LacZ* mice (Xu et al. 2008). Our observation that *Nkx2.1* expression disappears at the Dg after E13.5—a period when many supragranular interneurons are produced—may be relevant to explain these data. Early Dg-derived cells might be represented instead among the reporter-colabeled infragranular population (see Sousa et al. 2009). Xu et al. (2008) conjectured that the subpopulation of SST cortical interneurons that was not colabeled in their transgenic mice might derive from the dorsal portion of the MGE (corresponding to the pMGE1 domain; Fig. 1c), on the rationale that *Nkx2.1Cre* activity was practically absent in the pMGE1 domain, as opposed to distinct *Nkx6.2* expression. It was accordingly suggested that SST cells lacking β-gal reaction derive from pMGE1, whereas those that coexpress the reporter would derive from a different domain (Xu et al. 2008). This conclusion seemed consistent with earlier in utero fate-mapping studies (Xu et al. 2004; Butt et al. 2005). In any case, these data are also consistent with an origin of both early and late SST neurons within the Dg area (pMGE5), due to the observed local downregulation of *Nkx2.1* expression there.

After initial experimental reports showed that the MGE gives rise to PV and SST cortical interneurons (Xu et al. 2004; Butt et al. 2005; Ghanem et al. 2007), Flames et al. (2007) performed in utero transplantation of GFP-expressing MGE cells into isochronic host embryos at E13.5. They found that around 30 % of the GFP-positive cells coexpressed SST, as opposed to roughly 50 % of cells coexpressing PV. In the same report, a small cube of GFP-positive tissue obtained from the dorsal pMGE1 subdomain at E13.5 (the pallidal domain closest to the striatum; Fig. 1c) was dissociated, and the cells were grafted into the MGE of an isochronic host embryo. The distribution of GFP-expressing cells in the host mice at P14 revealed that over 60 % of transplanted cells were SST positive, against 7 % labeling obtained with similar grafts of pMGE4 cells; no experiment was performed with cells from our Dg, which corresponds to pMGE5 (Flames et al. 2007; compare Fig. 1c). The dorsal Pal domain was accordingly proposed as the main origin of the SST^+^/CR^+^ Martinotti cells, and this conclusion was corroborated by other authors (Flames et al. 2007; Fogarty et al. 2007; Wonders et al. 2008; see below). The concern raised by these apparently strong data, is that E13.5 may be a rather late stage for detecting the origin of *Sst* cells, since a substantial number of the corticopetal Dg-originated elements have probably slipped beyond the MGE at that stage; moreover, many migrating Dg elements must be present at the transient SSpM stream found crossing the pallidal marginal stratum at E13.5. Accordingly, the cells dissociated at the pMGE1 locus at E13.5 may have included a significant number of Dg-derived *Sst* neurons. The conclusion of Flames et al. (2007) as regards the pMGE1 origin of *Sst* cells thus appears weaker than expected, pending further experimental tests with younger pMGE1 cells (e.g., taken at E10.5), and including comparisons with the diagonal pMGE5 area. Nevertheless, though we saw little histologic evidence of a pMGE1 origin of *Sst* cells, our results do not allow us to exclude categorically that some *Sst* cells may arise at this pallidal subarea.

Wonders et al. (2008) dissociated dorsal and ventral GFP-positive Pal tissue at E13.5 and injected the cells into newborn cortex, checking their differentiation into SST versus parvalbumin (PV) cells. They concluded that the dorsal Pal was mainly implicated in the production of SST cortical interneurons (63 % SST versus 30 % PV), whereas relatively more interneurons expressing parvalbumin apparently originated at the ventral MGE (31 % SST versus 59 % PV). The location given for the ventral MGE in their Figure 2A is compatible with the topography of the Dg (the depicted explants most probably contained a deep part of the mantle zone, in our opinion). In order to eliminate potential passing cells, Wonders et al. (2008) explanted again dorsal Pal cells at E12.5 1 h after injecting BrdU to the dams and cultured them in vitro for 10 days. The rationale was to obtain in vitro-differentiated SST neurons double-labelled for GFP and BrdU, that is, derived exclusively from dorsal Pal progenitors They obtained over 60 % double-labeled SST cells for dorsal tissue versus 15 % for ventral tissue (their Fig. 3F). A concern that applies to these data is whether differentiation of dissociated immature cells injected directly into the newborn cortex, or in vitro differentiation of BrdU-labelled progenitors, reproduces faithfully enough the in vivo conditions for the relevant phenotypic decisions. Moreover, the first results may be contaminated by migrating Dg cells passing next to pMGE1 (as in Flames et al. 2007), and the in vitro results may result from re-specification of the explanted dorsal Pal progenitors (e.g., by downregulation of their *Nkx2.1* expression). Appropriate tests should be designed to check these possibilities. In any case, taken at face value, these results suggest that SST cortical interneurons are produced both at the dorsal and ventral Pal, that is at the pMGE1 and pMGE5 domains, thus providing partial support for our results. This suggests that our negative data about *Sst* cells originating within the Pal, or their absence inside the early Pal mantle layer, might be caused by late transcription of the *Sst* gene in the pMGE1-derived elements.

The homeodomain transcription factor *Nkx6.2* is expressed at the border between striatal and pallidal domains. It is specifically observed in a small subset of neural progenitors localized across the striatal progenitor domain pLGE4 and the pallidal progenitor domain pMGE1 (Stenman et al. 2003; Flames et al. 2007; Fogarty et al. 2007; Sousa et al. 2009). Genetic inducible fate-mapping using *Nkx6.2CreER/*+ mice showed EGFP-colabeling in SST-positive cortical interneurons (Sousa et al. 2009). Interestingly, the *Nkx6.2*-*Cre/R26R*-*GFP* transgenic mice line used by Fogarty et al. (2007) also showed GFP expression in scattered neuroepithelial cells located more ventrally in the caudal ventral MGE (the Dg area), suggesting that the results of Sousa et al. (2009) are not inconsistent with a double origin of SST cells. This work concluded that SST+/CR− cells generated at E10.5 selectively target deep cortical layers, whereas SST+/CR+ cells are generated at E12.5 and invade superficial cortical layers. The authors did not mention whether cells from this lineage differentiate within subpallial formations after radial migration.

Fogarty et al. (2007) concluded that only small subsets of the interneurons produced in their *Nkx6.2*-*Cre/R26R*-*GFP* transgenic mice line coexpress GFP and the studied interneuron markers (including SST; their Figure 3A–D). This generates the possibility that most SST cells are derived from other pallidal domains, including the Dg. When *Nkx6.2*-*Cre/Nkx2.1*-*Cre/R26R*-*GFP* double transgenic mice (lineage tracing targeting all MGE and POA derivatives) were studied, the majority of SST+ cells (70–80 % of cortical motor and somatosensory SST cells) were marked with GFP. Since *Nkx2.1*Cre activity is practically absent at the pMGE1 domain (Fogarty et al. 2007; Xu et al. 2008). This result suggests that most SST-positive cells are generated in the alternative source, the ‘caudal ventral MGE’. i.e., the Dg (Fogarty et al. 2007). Approximately 35 % of *Nkx2.1Cre*-*GFP*-expressing cells were SST positive and they represent around 70–80 % of this type of interneuron in the motor and somatosensory cortex (Fogarty et al. 2007).

Poor recombination of R26R-GFP with *Lhx6*-*Cre* was also observed at the pMGE1 (Fogarty et al. 2007). Using both *Nkx2.1Cre:R26R*-*GFP* and *Lhx6Cre:R26R*-*YFP*, these authors thus essentially identified the origin of most SST interneurons as corresponding to the ‘caudal ventral MGE’, which represents the Dg domain. *Lhx6Cre:R26R*-*YFP* transgenic mice displayed in the motor and somatosensory cortex 100 % of SST-positive cells labeled with YFP, as well as nearly 100 % of the PV+ and CB+ interneurons. A high degree of SST and *Lhx6* coexpression was also observed using *Lhx6*^+/LacZins^ mice, and *Sst*-expressing cells were drastically reduced (around 93 %) in *Lhx6*^−/−^ mutants (Liodis et al. 2007, Zhao et al. 2003). These experimental approaches suggest strongly that the ‘caudal ventral MGE’ (Dg) domain contains the main SST-cell source; early activity of the genes *Nkx2.1* and *Lhx6* seems required to generate SST-positive interneurons (Sussel et al. 1999; Liodis et al. 2007).

Carney et al. (2010) analyzed the lineage origin of medial amygdala components, immunolabeling SST cells in combination with an anti-β-galactosidase (β-gal) antibody to visualize nuclear staining in recombined cells from *Nkx2.1*^*Cre*^, *Shh*^*Cre*^ and *Gli1*^*CreER(T2)*^*:Tau*^*mGFP*^ brains. This analysis revealed that 10 and 23 % of *Nkx2.1*^*Cre*^*:Tau*^*mGFP*^ and 20 and 24 % of *Gli1*^*CreER(T2)*^*:Tau*^*mGFP*^ recombined cells coexpressed SST at the medial posterodorsal and medial posteroventral nuclei (MePD, MePV) of the amygdala, respectively. This proportion was reduced to 2 and 3 % of cells coexpressing SST and *Shh*^*Cre*^*:Tau*^*mGFP*^ in the MePD and MePV, respectively (Carney et al. 2010). These results led to the conclusion that SST cells that populate the medial amygdala derive from the ‘caudal ventral MGE’, that is, the Dg.

Flandin et al. (2010) used *Shh*-*Cre* mice and a floxed *Nkx2.1* allele to selectively knock-out *Nkx2.1* function from the *Shh*-expressing subpallium (POA1 ventricular zone and *Shh*-positive migrated cells in the pallidal mantle; eventually, also some ventricular-cell patches in Dg). They observed that the globus pallidus cell population was substantially eliminated, whereas most cortical and striatal interneurons were generated, excepting the striatal cholinergic neurons. In their Discussion, the authors deduce that pMGE1-3 (perhaps also pMGE4) generate most parvalbumin+ and somatostatin+ neo-cortical and hippocampal interneurons, because these domains do not express *Shh*. According to our interpretation, the *Sst* cells originated from the Dg ventricular domain—the pMGE5 area—may have partly escaped the targetted knock-out, since *Shh* expression only appears there in a patchy fashion (Figs. 4, 5, 6, S1; note Flandin et al. 2010 themselves observed reduced integration of *ShhCre* activity in the Dg domain in *ROSA;Shh*^*Cre/*+^ mice). A 40 % of reduction in cortical SST+ cells was observed in the *ShhCre*-*Nkx2.1*-floxed mutant (Flandin et al. 2010), consistently with the possibility that this represents the product of a Dg progenitor fraction (40 %) that expresses *Shh,* whereas the remaining SST+ elements may be originated by the *Shh*-negative Dg progenitors, and are thus unaffected. On the other hand, a strong knock-out effect probably occurred at the septal Dg/POA sectors, where *Shh* signal is intense in the ventricular zone (Figs. 4, 5, 6, S1); this is the place where striatal cholinergic neurons are perhaps produced (García-López et al. 2008; Hoch et al. 2015). The reported high activation of *ShhCre* in several pallidal domains is difficult to understand, because *Shh* is not expressed at all in the pallidal ventricular zone (present results); the missing or reduced globus pallidus phenotype may be due to a strong knock-out effect at the POA1 area, whose ventricular zone expresses strongly *Shh* and produces *Shh*-positive neurons that migrate into the pallidal mantle. These migrated cells may release SHH, which might be required for the normal development of the globus pallidus (e.g., maintenance of Nkx2.1 activity).

Most of these in vivo and in vitro fate-mapping and transgenic mice studies thus suggest a principal ‘ventral MGE’ main source of *Sst* cells, consistent with our Dg area, highlighted in our in situ analysis as the main origin of this interneuronal type. Our study emphasizes the supra- and subcapsular histogenetic and topographic unity of the Dg domain along the septoamygdaloid axis, where various radially migrated derivatives are established (diagonal complex/medial septum, BSTM, amygdaloid BST and CeL nuclei), and from where laterally oriented tangential migrations proceed into the striatum and the whole pallium (sidestepping via the SSpM the apparently non-permissive or repelling pallidum). Several of the cited studies proposed that GABAergic interneurons from the MGE (including SST cells) first invade the pallium around E12.5 (e.g., Corbin et al. 2001; Marin and Rubenstein 2003); our sequential analysis suggests that this process already starts at E10.5 out of the Dg domain, and pioneering cells already reach the cortex around E12.5.

#### Preoptic origins

The POA region forms the ventromedial *Nkx2.1*-positive component of the MGE. It shows three dorsoventrally superposed progenitor domains, identified as POA1 (next to Dg), POA2 (next to the terminal lamina) and POH (preopto-hypothalamic transition area, limiting with the paraventricular alar hypothalamus) (Flames et al. 2007; Bardet et al. 2010; Medina and Abellan 2012; Puelles et al. 2012, 2013). Lineage-tracing experiments using intra-utero electroporation of a *Nkx5.1Cre*:R26R-YFP construct demonstrated labeled preoptic GABAergic cells that migrate tangentially into the cortex, septum, striatum, and amygdala (Gelman et al. 2009). These cells frequently coexpressed NPY, but never SST. Moreover, they derive from a *Nkx2.1*+*/Lhx6*- lineage, which confirms their POA identity (Gelman et al. 2009). Preoptic *Nkx5.1* signal present in postmitotic cells was initially held to be a general POA marker (Wang et al. 2000; Gelman et al. 2009), but Gelman et al. (2011) later acknowledged that these cells originate specifically at the *Shh*-positive POA1 domain, as is corroborated by our present data (see Fig. 6); indeed, we found that this restricted origin includes the median or acroterminal part of pPOA1, which encompasses the terminal lamina (Puelles et al. 2012). Gelman et al. (2011) stated that the *Nkx5.1*-positive elements that migrate out of the pPOA1 area rapidly lose this expression. Comparison of *Nkx5.1* expression with *Shh* expression in the subpallial mantle indicated that preoptic *Shh*-expressing cells are likewise produced selectively within the pPOA1 area, from where they selectively migrate tangentially into the pallidum (after crossing the Dg domain; note we never saw them entering the striatal mantle). In the chick and mouse, the pPOA2 area expresses *Nkx2.1*, but not *Shh* (Bardet et al. 2010; Flandin et al. 2010). Gelman et al. (2011) investigated a subarea of pPOA2 that expresses *Dbx1*, from where a separate population of derived GABAergic cells migrate into the cortex, invading predominantly its deep layers. Phenotypic analysis of these cells at P14 suggested that nearly 50 % of the cortical preoptic-derived *Dbx1* cells contain PV (the authors comment that the proportion may be larger, though, since this marker is a late differentiating one, and many of the observed GABAergic *Dbx1*-derived cells did not express any peptidic markers) and approximately 25 % contained SST. They deduced that both PV and SST cells are produced at the pPOA2 area. This observation is contradictory with our observations, because we never saw *Sst* cells arising within the preoptic area. We would thus predict that the SST elements observed by Gelman et al. (2011) most probably originated likewise in the Dg area. Primary evidence that can be adduced in favor of this interpretation is the expression of *Dbx1* mapped at E11.5 in the Allen Developing Mouse Brain Atlas (http://www.developingmouse.brain-map.org); the available sagittal and coronal sections both illustrate that at this stage the expression domain of *Dbx1* extends more importantly across Dg than across the preoptic area. Part of the *Dbx1*-derived cells may be thus diagonal in character, rather than preoptic. Gelman et al. (2011) further acknowledged that permanent tracing of *Dbx1*-derived cells in Dbx1^Cre^; ROSA26^YFP^ embryos generated ‘a few clones of YFP-expressing cells… in the MGE’. The authors checked the relationship of *Dbx1*-derived cells with *Lhx6*-derived cells (*Lhx6* is a general MGE marker excluded from the POA; Liodis et al. 2007); the results indicated that some 36 % of the cortical *Dbx1*-derived cells co-express *Lhx6*, confirming that their origin can not be purely preoptic. On the other hand, Fogarty et al. (2007) concluded that 100 % of cortical SST cells express *Lhx6*. This suggests that the 25 % of SST neurons counted by Gelman et al. (2011) among the cortical *Dbx1* progeny may originate as we suggest within the *Dbx1*+/*Lhx6*+ Dg sector of the MGE.

To summarize, in our opinion the published experimental evidence on lineage-tracing of SST-positive cells seems somewhat vague and inconclusive about their precise origin within the MGE, partly due to 1) confusion in the denomination and/or tracing of the relevant progenitor areas, 2) experimental study mainly of suboptimal stages (e.g., E13.5) which allow already migrated, or migrating neurons, to be ascribed to a wrong source, and 3) insufficient use (or dearth) of efficient molecular delimitation criteria for the Pal, Dg and POA subpallial domains and their diverse septoamygdaloid sectors. The present descriptive analysis cannot correct all these problems, but provides a solid basis of data that must be made consistent with any experimental analysis, as long as the existence of invisible *Sst* cells is not demonstrated beyond any doubt. They also illuminate in a novel way the development of the BSTM, CeL, and globus pallidus formations, as well as the intraamygdaloid relationships of the main subpallial domains.

## Electronic supplementary material

Below is the link to the electronic supplementary material.

**Fig.S1** - Rostrocaudal series of topologically transversal cryostat sections through the MGE (see plane in Fig. 1 **a**) at E10.5, illustrating in correlative adjacent sections the topography of *Sst* cells relative to *Shh* and *Lhx7*-*8* expression: (**a-f**) *Sst*; (**g-l**) *Shh*; (**m-r**) *Lhx7*-*8*. Early *Sst* cells are observed close to the Dg ventricular zone (**c,d**). They aggregate at the marginal pallidal stratum and start to invade the striatum (Pal, St; **a-f**). The Dg ventricular zone shows patchy *Shh* expression (**g-l**). *Lhx7*-*8* cells are clearly produced both at the pallidum and diagonal domains, but apparently not at the preoptic area (**n-r**) (TIFF 17717 kb)

**Fig.S2** – Sagittal section through the telencephalic subpallium in an E14.5 embryo (**a**) and two details at higher magnification, illustrating the topographic relationship of the diagonal *Sst*-positive supracapsular periventricular BSTM derivatives with DLX5 (**b**) and OTP (**c**) immunoreaction (superposed images from adjacent sections). (**a**) White dash lines delimit the St, Pal and Dg domains. The marker DLX5 labels uniformly the striatum, pallidum and diagonal, with a sharp boundary relative to the underlying alar peduncular hypothalamus (PHy), traversed vertically by the fibers of the cerebral peduncle (ped). The supracapsular BSTM (BSTMsc) is strongly *Sst*-positive, and lies at the border of the DLX5-positive field (detail at higher magnification in **b**). (**c**) shows a digital superposition of the *Sst* image with an adjacent section immunoreacted for OTP; this labels a distinct periventricular OTP-positive population that corresponds to the supracapsular migratory stream of *Otp* cells that move from the paraventricular alar hypothalamic area towards the medial amygdala; the comparison indicates that the latter migration courses outside the subpallium, that is, penetrates directly the pallium at the telencephalic stalk, neighboring the Dg domain (TIFF 6885 kb)

**Fig.S3** - Sagittal (**a**) and horizontal (**b**) sections through the adult mouse telencephalon, reacted by ISH for *Sst*, and counterstained with tyrosine hydroxylase immunoreaction (TH), illustrating *Sst* cell distribution in the striatum, olfactory cortex, hippocampus and the amygdaloid area, where the CeL subnucleus of CA stands out by its dense somatostatinergic population. This is held to derive radially from the amygdaloid sector of the Dg subpallial domain, jointly with the amygdaloid nucleus of the BSTM complex (BSTMa; **b**). There are sparser populations in the medial and capsular parts of the CA (CeM, CeC). Note also a dispersed population of *Sst* cells in the striatum, while *Sst* cells in layer III of the olfactory cortex (PirCx) are abundant (**a,b**), similarly as in the medial amygdala (MA), basomedial nucleus (BM) and lateral amygdalar nuclesu (L). In contrast, the basolateral nucleus remains poorly populated by *Sst* cells (**a,b**). Finally, note hippocampal *Sst* cells in **a**, mainly at the subiculum (Sub), alveus of field CA1, stratum oriens, pyramidal layer and stratum lucidum of CA3, and the dentate hylus (DG) (TIFF 8203 kb)

**Fig.S4** – Plate illustrating RC2 immunoreactive radial glia in the stria terminalis region (st) of an E18.5 mouse embryo (80 μm-thick Vibratome sections). The section plane is oblique (opening approximately 45 degrees away from the midsagittal plane caudalwards), and courses along the septoamygdaloid axis (Se, A), in order to intersect longitudinally the stria terminalis tract (the cortical structure seen above the st-complex is the hippocampus). Three adjacent section planes are shown (**a-c**), which progress from medial (Dg domain) to lateral (Pal domain). Reference landmarks visible include the anterior commissure (ac; note it starts to divide into anterior and posterior components in **c**), the internal capsule (ic; it expands as it penetrates the globus pallidum, Pal), the diagonal band (db) and the nucleus of the lateral olfactory tract (NLOT). Pannels (**d,e**) show higher magnification views of the boxed areas in **b** and **c**. The central amygdalar region lies under the amygdalar end of the stria terminalis, accompanied by the amygdalar BST nucleus (Ce, BSTa). Radial glia fibres can be seen to originate at the ventricular lining (uppermost arrows in **d,e**), traverse vertically or obliquely the Ce field and end subpially in the neighborhood of the NLOT (other arrows in **b-e**). Some glial fibers are stretched along the stria terminalis (under the st label in **d,e**), probably a passive deformation caused by the growth of the internal capsule, and recuperate a vertical orientation as soon as they reach the Ce region. Radial glia fibres pertaining to the pallial amygdala adopt slightly different courses (A, BL; **a-e**) (TIFF 16425 kb)

## Abbreviations

A: Amygdala
AA: Anterior amygdala
ac: Anterior commissure
Acb: Accumbens nucleus
AH: Anterior lobe of hypophysis
AHi: Amygdalo-hippocampal area
Amygd: Amygdala
AOA: Anterior olfactory area
APi: Amygdaloid piriform area
B: Basal magnocellular nucleus of Meynert
BEC: Bed nucleus of external capsule
BL: Basolateral amygdaloid nucleus
BM: Basomedial amygdaloid nucleus
bp: Basal plate
BST: Bed nucleus of the stria terminalis
BSTa: Amygdaloid BST nucleus
BSTL: Lateral (pallidal) part of BST
BSTLsc: Supracapsular part of BSTL
BSTM: Medial (diagonal) part of BST
BSTMa: Anterior part of BSTM
BSTMsc: Supracapsular part of BSTM
BSTMps: Paraseptal part of BSTM
C: Central region of the subpallium
Ce: Central amygdaloid nucleus
CeC: Capsular part of Ce
CeL: Lateral part of Ce
CeM: Medial part of Ce
CGE: Caudal ganglionic eminence
CL: Claustrum
CL/I: Claustro-insular complex
CP: Cortical plate
CPu: Caudate putamen
Cx: Cortex
DB: Diagonal band nucleus
db: Diagonal band
Dg: Subpallial diagonal domain
DgA: Amygdaloid diagonal area
DgC: Central diagonal area
DgMC: Magnocellular diagonal nucleus
DgSe: Diagonal septum
EGP: Globus pallidus, external segment
ERh: Entorhinal cortex
FCx: Frontal cortex
GP: Globus pallidus
Hb: Habenula
HDB: Diagonal band, horizontal nucleus
Hi: Hippocampus
hp1: Hypothalamic prosomere 1
hp2: Hypothalamic prosomere 2
Hy: Hypothalamus
ic: Internal capsule
ICx: Insular cortex
ILCx: Infralimbic cortex
IGP: Globus pallidus, internal segment
IPAC: Intersticial nucleus of the posterior limb of the anterior commisure
L: Lateral amygdalar nucleus
LGE: Lateral ganglionic eminence
lot: Lateral olfactory tract
lt: Lamina terminalis
lv: Lateral ventricle
MA: Medial amygdala
MePV: Medial posteroventral nucleus of the amygdala
MGE: Medial ganglionic eminence
NCX: Neocortex
NLOT: Nucleus of the lateral olfactory tract
NLOTm: Migration stream of the nucleus of the lateral olfactory tract
OA: Olfactory amygdala
OB: Olfactory bulb
OCx: Orbitary cortex
os: Optic stalk
ot: Optic tract
OT: Olfactory tuberculum
OtpMS: *Otp*-positive cells migratory stream
p2: Prosomere 2
p3: Prosomere 3
Pa: Paraventricular hypothalamic area
Pal: Pallidum
PalSe: Pallidal septum
PallA: Pallial amygdala
ped: Peduncle
PHy: Peduncular hypothalamus
PirCx: Piriform cortex
PirCx III: Layer III of PirCx
PLCo: Posterolateral cortical amygdaloid nucleus
PMCo: Posteromedial cortical amygdaloid nucleus
POA: Preoptic area
POA1: Area 1 of POA
POA2: Area 2 of POA
POASe: Preoptic septum
POH: Preopto-hypothalamic transition area
PT: Pretectum
PSe: Paraseptal subpallium
PTh: Prethalamus
PThE: Prethalamic eminence
Rh: Rhombencephalon
S: Subiculum
Se: Septum
SeDg: Septo-diagonal area (paraseptal diagonal domain)
SePal: Septo-pallidal area (paraseptal pallidal domain)
SI: Substantia innominata
SSpM: Superficial subpallial migration stream
slt: Sulcus terminalis
st: Stria terminalis
St: Striatum
StSe: Striatal septum
Subpial migr: Subpial migratory pathway
Subvent migr: Subventricular migratory pathway
SvSpM: Subventricular subpallial migratory stream
Tel: Telencephalon
Th: Thalamus
THy: Terminal hypothalamus
VPal: Ventral pallidum
VSt: Ventral striatum
vz: Ventricular zone
Zli: Zona limitans interthalamica
