# Supplementary figures and images for: Radial and tangential migration of telencephalic somatostatin neurons originated from the mouse diagonal area
Source: Brain Struct Funct. 2015 Jul 19;221:3027–65. doi: 10.1007/s00429-015-1086-8 (PMC4920861; doi:10.1007/s00429-015-1086-8)

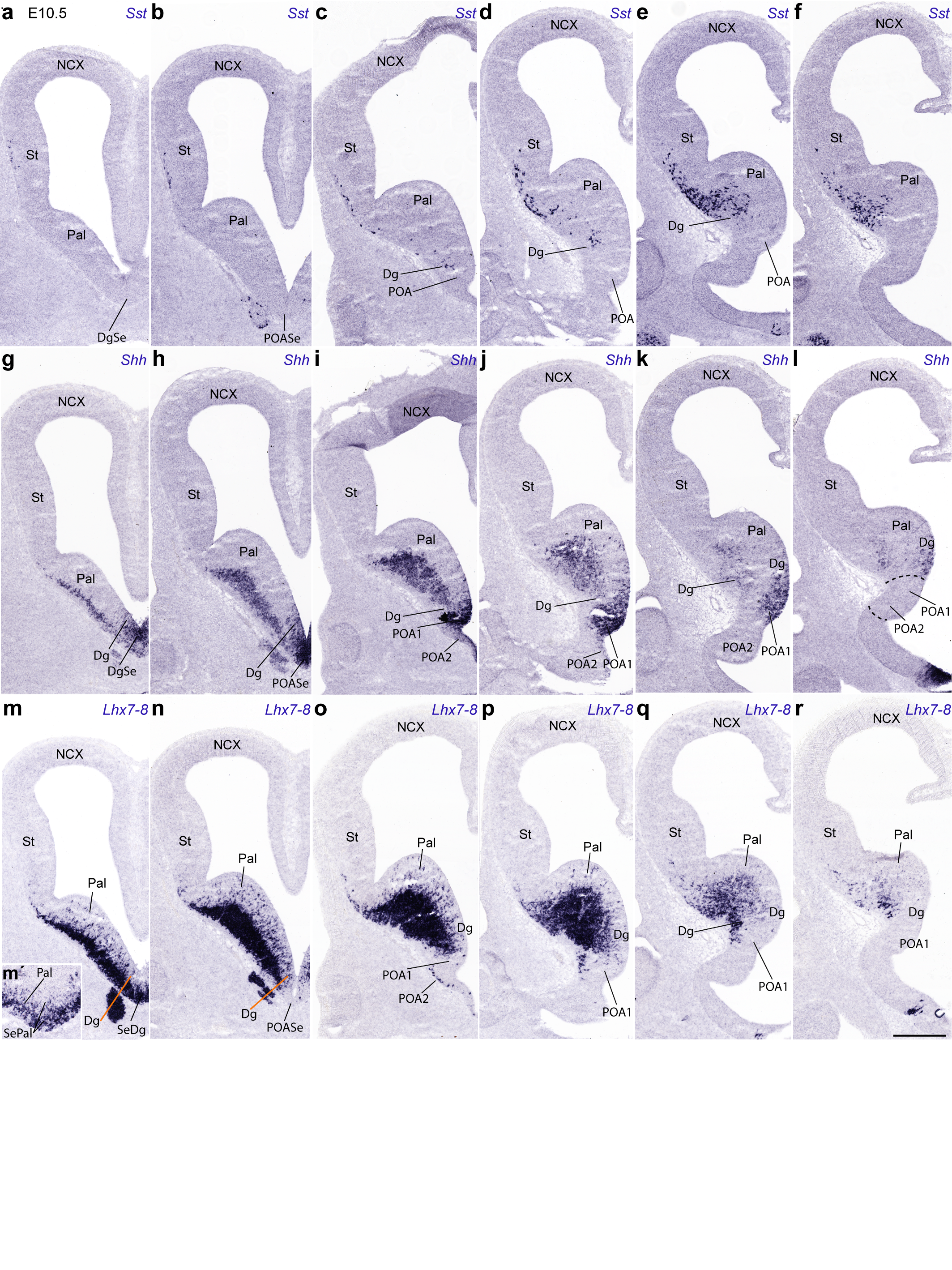

Supplement: Supplementary file 1 — Fig.S1 - Rostrocaudal series of topologically transversal cryostat sections through the MGE (see plane in Fig. 1 a) at E10.5, illustrating in correlative adjacent sections the topography of Sst cells relative to Shh and Lhx7-8 expression: (a-f) Sst; (g-l) Shh; (m-r) Lhx7-8. Early Sst cells are observed close to the Dg ventricular zone (c,d). They aggregate at the marginal pallidal stratum and start to invade the striatum (Pal, St; a-f). The Dg ventricular zone shows patchy Shh expression (g-l). Lhx7-8 cells are clearly produced both at the pallidum and diagonal domains, but apparently not at the preoptic area (n-r) (TIFF 17717 kb) [file 429_2015_1086_MOESM1_ESM.tif]

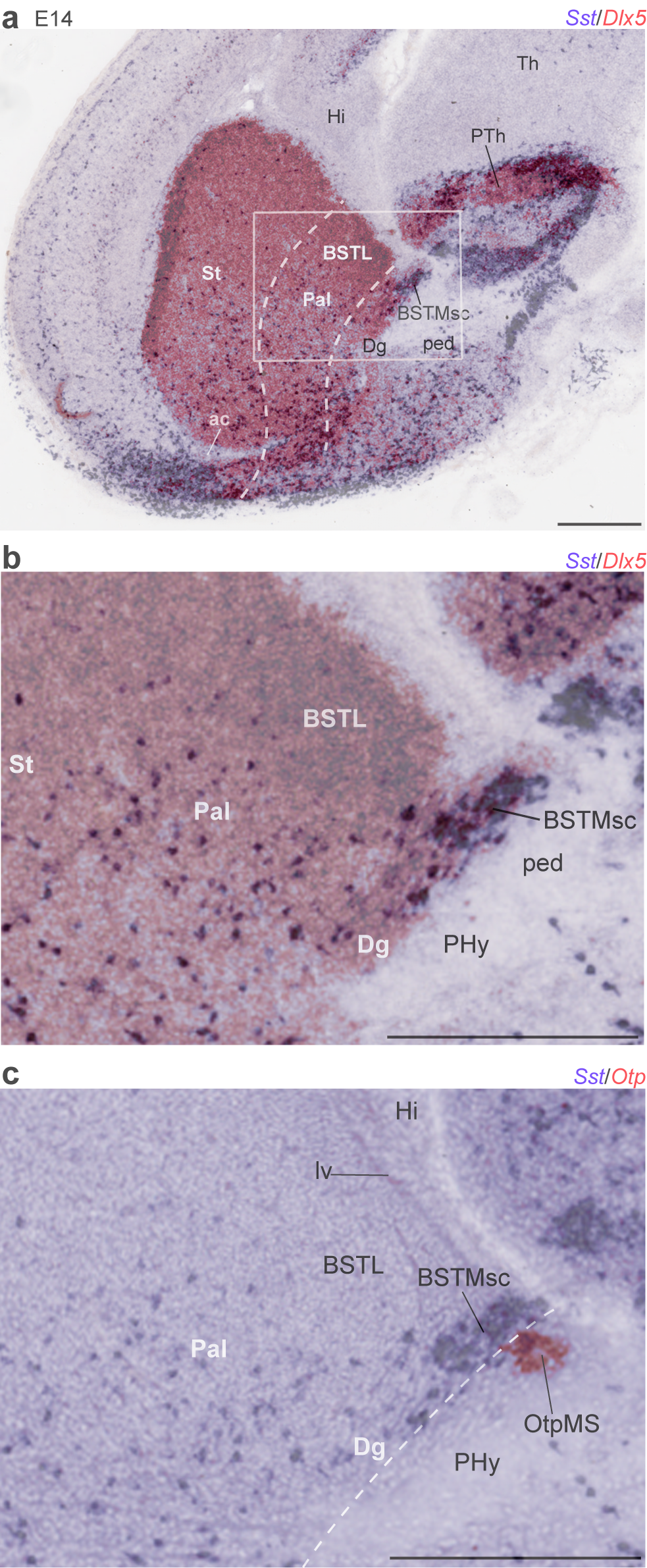

Supplement: Supplementary file 2 — Fig.S2 – Sagittal section through the telencephalic subpallium in an E14.5 embryo (a) and two details at higher magnification, illustrating the topographic relationship of the diagonal Sst-positive supracapsular periventricular BSTM derivatives with DLX5 (b) and OTP (c) immunoreaction (superposed images from adjacent sections). (a) White dash lines delimit the St, Pal and Dg domains. The marker DLX5 labels uniformly the striatum, pallidum and diagonal, with a sharp boundary relative to the underlying alar peduncular hypothalamus (PHy), traversed vertically by the fibers of the cerebral peduncle (ped). The supracapsular BSTM (BSTMsc) is strongly Sst-positive, and lies at the border of the DLX5-positive field (detail at higher magnification in b). (c) shows a digital superposition of the Sst image with an adjacent section immunoreacted for OTP; this labels a distinct periventricular OTP-positive population that corresponds to the supracapsular migratory stream of Otp cells that move from the paraventricular alar hypothalamic area towards the medial amygdala; the comparison indicates that the latter migration courses outside the subpallium, that is, penetrates directly the pallium at the telencephalic stalk, neighboring the Dg domain (TIFF 6885 kb) [file 429_2015_1086_MOESM2_ESM.tif]

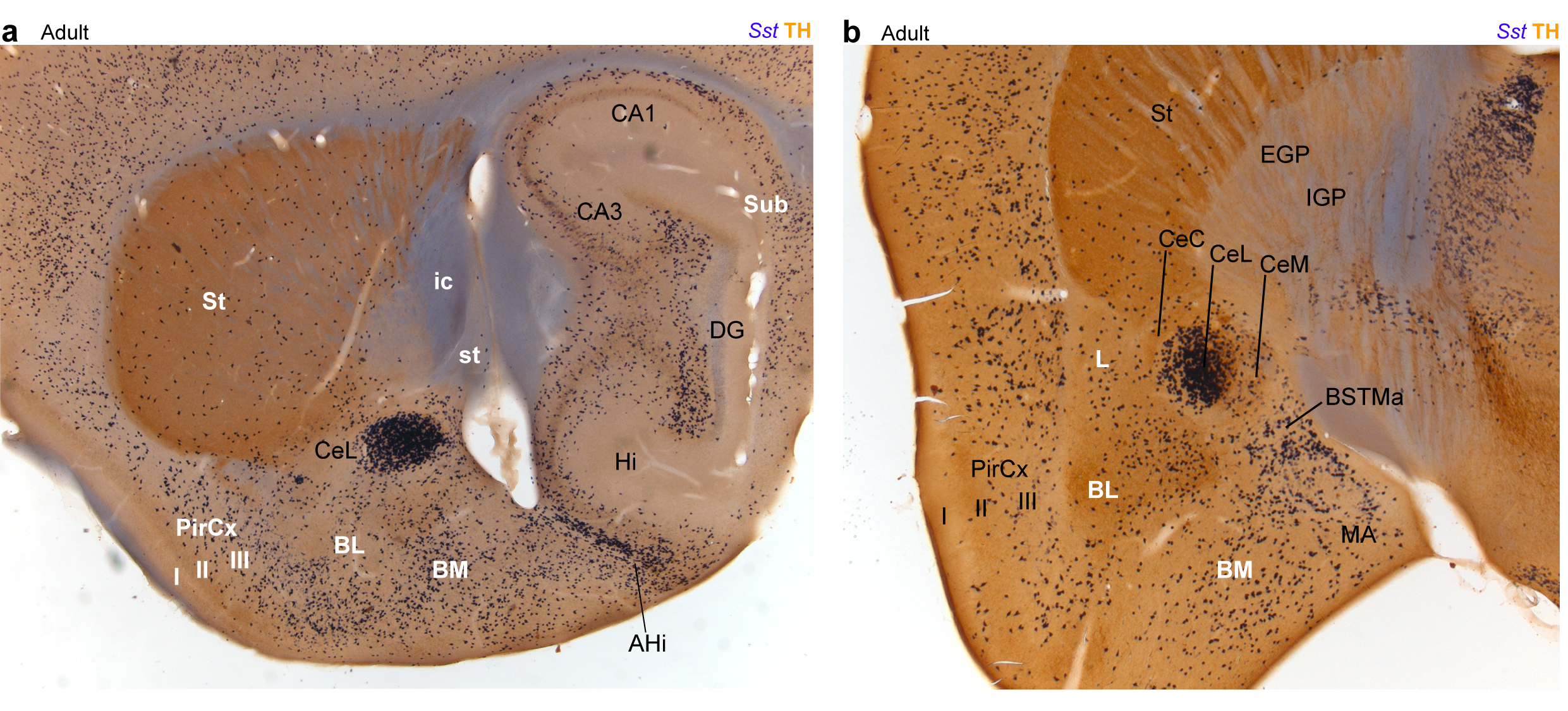

Supplement: Supplementary file 3 — Fig.S3 - Sagittal (a) and horizontal (b) sections through the adult mouse telencephalon, reacted by ISH for Sst, and counterstained with tyrosine hydroxylase immunoreaction (TH), illustrating Sst cell distribution in the striatum, olfactory cortex, hippocampus and the amygdaloid area, where the CeL subnucleus of CA stands out by its dense somatostatinergic population. This is held to derive radially from the amygdaloid sector of the Dg subpallial domain, jointly with the amygdaloid nucleus of the BSTM complex (BSTMa; b). There are sparser populations in the medial and capsular parts of the CA (CeM, CeC). Note also a dispersed population of Sst cells in the striatum, while Sst cells in layer III of the olfactory cortex (PirCx) are abundant (a,b), similarly as in the medial amygdala (MA), basomedial nucleus (BM) and lateral amygdalar nuclesu (L). In contrast, the basolateral nucleus remains poorly populated by Sst cells (a,b). Finally, note hippocampal Sst cells in a, mainly at the subiculum (Sub), alveus of field CA1, stratum oriens, pyramidal layer and stratum lucidum of CA3, and the dentate hylus (DG) (TIFF 8203 kb) [file 429_2015_1086_MOESM3_ESM.tif]

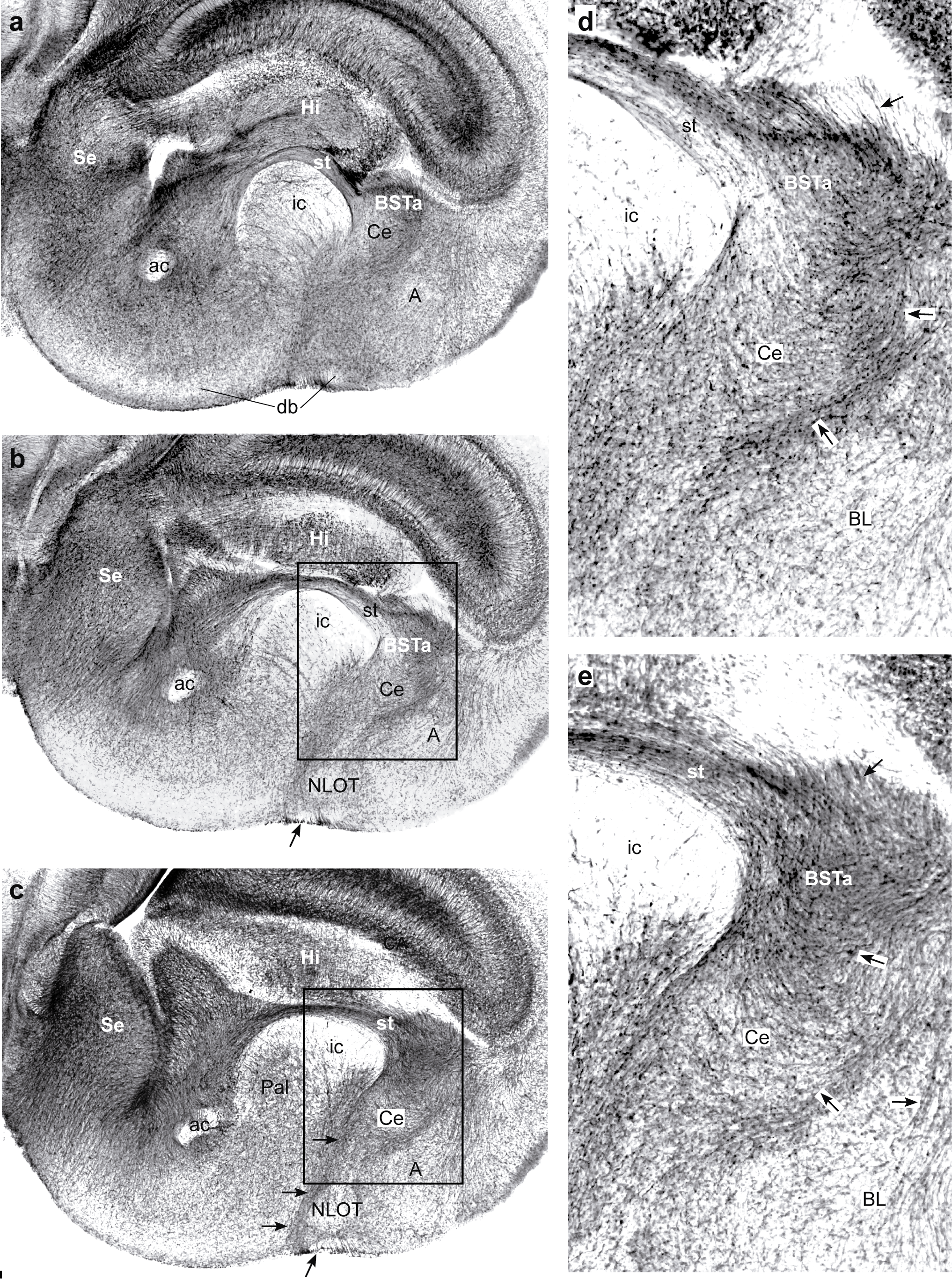

Supplement: Supplementary file 4 — Fig.S4 – Plate illustrating RC2 immunoreactive radial glia in the stria terminalis region (st) of an E18.5 mouse embryo (80 μm-thick Vibratome sections). The section plane is oblique (opening approximately 45 degrees away from the midsagittal plane caudalwards), and courses along the septoamygdaloid axis (Se, A), in order to intersect longitudinally the stria terminalis tract (the cortical structure seen above the st-complex is the hippocampus). Three adjacent section planes are shown (a-c), which progress from medial (Dg domain) to lateral (Pal domain). Reference landmarks visible include the anterior commissure (ac; note it starts to divide into anterior and posterior components in c), the internal capsule (ic; it expands as it penetrates the globus pallidum, Pal), the diagonal band (db) and the nucleus of the lateral olfactory tract (NLOT). Pannels (d,e) show higher magnification views of the boxed areas in b and c. The central amygdalar region lies under the amygdalar end of the stria terminalis, accompanied by the amygdalar BST nucleus (Ce, BSTa). Radial glia fibres can be seen to originate at the ventricular lining (uppermost arrows in d,e), traverse vertically or obliquely the Ce field and end subpially in the neighborhood of the NLOT (other arrows in b-e). Some glial fibers are stretched along the stria terminalis (under the st label in d,e), probably a passive deformation caused by the growth of the internal capsule, and recuperate a vertical orientation as soon as they reach the Ce region. Radial glia fibres pertaining to the pallial amygdala adopt slightly different courses (A, BL; a-e) (TIFF 16425 kb) [file 429_2015_1086_MOESM4_ESM.tif]
